# Supplementary material for: The Global, Regional and National Burden of Pancreatic Cancer Attributable to Smoking, 1990 to 2019: A Systematic Analysis from the Global Burden of Disease Study 2019
Source: Int J Environ Res Public Health. 2023 Jan 14;20(2):1552. doi: 10.3390/ijerph20021552 (PMC9859604; doi:10.3390/ijerph20021552)
Supplement: Supplementary file 1 [file ijerph-20-01552-s001.zip › ijerph-2054937-supplementary.pdf]

Supplementary Data

Title: The global, Regional and National Burden of Pancreatic Cancer Attributable to Smoking, 1990 to 2019: A Systematic Analysis from the Global Burden of Disease Study 2019

Supplementary File S1: GBD 2019 Overview

Supplementary File S2: Smoking relative risk of pancreatic cancer in GBD 2019

Supplementary File S3 Sociodemographic index

Supplementary File S4 Joinpoint regression model

Supplementary File S5 Supplementary figures

Figure S1 Age-specific counts of deaths (A) and DALYs (B) of pancreatic cancer attributable to smoking by sex, 2019.

Figure S2 The trend in the proportion of deaths and DALYs attributable to smoking from 1990 to 2019. (A) Deaths; (B) DALYs.

Figure S3 The proportion of age-specific deaths attributable to smoking for (A) males and (B) females in 2019.

Figure S4 The change trend in the age-standardized DALY rate of pancreatic cancer attributable to smoking in four continents, 1990 to 2019. (A) Africa; (B) America; (C) Asia; (D) Europe.

Figure S5 Cluster plot of countries/territories with the same AAPC.

Figure S6 The correlation between AAPCs (1990 to 2019) and ASRs (in 2019), and HDI (in 2019).

Figure S7 The change trend in ASDR of pancreatic cancer attributable to smoking in five SDI quintiles, 1990 to 2019. (A) High SDI; (B) high-middle SDI; (C) middle SDI; (D) low-middle SDI; (F) low SDI.

Figure S8 The change trend in the age-standardized DALY rate of pancreatic cancer attributable to smoking in five SDI quintiles, 1990 to 2019. (A) High SDI; (B) high-middle SDI; (C) middle SDI; (D) low-middle SDI; (F) low SDI.

Figure S9 The trend in the age-standardized DALY rate of smoking-related pancreatic cancer across 21 GBD regions by SDI, from 1990 to 2019

Figure S10 The relationship between the age-standardized DALY rate of smoking -related pancreatic cancer and SDI among all countries/territories in 2019

Supplementary File S6: Supplementary tables

Table S1 DALYs and age-standardized DALY rate of smoking-attributable pancreatic cancer among all regions, 1990 to and 2019

Table S2 Death and its change trends in smoking-related pancreatic cancer from 1990 to 2019 among all countries/territories

Table S3 The DALY and its change trends in smoking-related pancreatic cancer from 1990 to 2019 among all countries/territories

Supplementary File S1 GBD 2019 Overview

1. The Global Burden of Diseases 2019 (GBD 2019)

GBD 2019 is a collaborative research effort aimed at estimating worldwide population, fertility, morbidity, and mortality. GBD draws on the expertise of an extensive collaborator network from around the world. All data contained 369 diseases and injuries and their 87 risk factors in all regions and countries and territories.

2. GBD estimate and measure

The latest version of the data download tool contains core summary results for GBD 2019 (https://vizhub.healthdata.org/gbd-results). The evaluation indicators in GBD 2019 include cause of death or injury, risk factor, etiology, impairment, health-adjusted life expectancy, population, fertility and all-cause mortality. In this study, we mainly used the estimate of risk factors. The measure of c risk factors includes deaths, disability-adjusted life years (DALYs), years lived with disability (YLDs) and years of life lost (YLL).

3. Information classification

The GBD 2019 risk factor and cause list are organized into a hierarchy (four levels). Level 1 contains 3 groups, named “Environmental/occupational risk”, “Behavioral risk” and “Metabolic risk”. For cause list, it contains 3 groups, named “Communicable, maternal, neonatal and nutritional diseases”, “Non-communicable diseases” and “Injuries”. The “Behavioral risk” includes nine risk factors: “Child and maternal malnutrition”, “Tobacco”, “Alcohol use”, “Drug use”, “Dietary risks”, “Intimate partner violence”, “Unsafe sex”, “Childhood sexual abuse and bullying” and “Low physical activity”. In this study, we first selected “Tobacco” in the risk factor list. Then, “Pancreatic cancer” was selected in the list of “Neoplasms” in the “Non-communicable diseases”. Estimates for the GBD 2019 cover the data from 1990 to 2019 for both sexes, male and female. Age information is presented as 5-year age

groups. Age-standardized information is also provided.

4. Data analysis

The age-standardized rate is a measure that can eliminate the influence of population age structure differences to the greatest extent. ASRs were calculated on the basis of the following formula:

$$ASR = \sum_{i=1}^{A} a_i w_i \Bigg/ \sum_{i=1}^{A} w_i \times 100{,}000$$

DALYs were also defined as years of healthy life lost, estimated by the sum of the YLDs and the YLLs. YLDs are calculated by multiplying the number of incident cases, disability weight and average duration of disability years. YLLs are the multiplication of the number of deaths and standard life expectancy at the age of death in years.

Supplementary File S2 Smoking relative risk of pancreatic cancer in GBD 2019

Risk - Outcome	Category / Units	Morbidity / Mortality	Sex	30-34 years	35-39 years	40-44 years	45-49 years	50-54 years	55-59 years	60-64 years	65-69 years	70-74 years	75-79 years	80-84 years	85-89 years	90-94 years	95+ years
**Smoking**																	
Pancreatic cancer	0 Pack Years	Both	Female	1.00 (1.00 to 1.00)	1.00 (1.00 to 1.00)	1.00 (1.00 to 1.00)	1.00 (1.00 to 1.00)	1.00 (1.00 to 1.00)	1.00 (1.00 to 1.00)	1.00 (1.00 to 1.00)	1.00 (1.00 to 1.00)	1.00 (1.00 to 1.00)	1.00 (1.00 to 1.00)	1.00 (1.00 to 1.00)	1.00 (1.00 to 1.00)	1.00 (1.00 to 1.00)	1.00 (1.00 to 1.00)
Pancreatic cancer	0 Pack Years	Both	Male	1.00 (1.00 to 1.00)	1.00 (1.00 to 1.00)	1.00 (1.00 to 1.00)	1.00 (1.00 to 1.00)	1.00 (1.00 to 1.00)	1.00 (1.00 to 1.00)	1.00 (1.00 to 1.00)	1.00 (1.00 to 1.00)	1.00 (1.00 to 1.00)	1.00 (1.00 to 1.00)	1.00 (1.00 to 1.00)	1.00 (1.00 to 1.00)	1.00 (1.00 to 1.00)	1.00 (1.00 to 1.00)
Pancreatic cancer	10 Pack Years	Both	Female	1.86 (1.61 to 2.11)	1.86 (1.61 to 2.11)	1.86 (1.61 to 2.11)	1.86 (1.61 to 2.11)	1.86 (1.61 to 2.11)	1.86 (1.61 to 2.11)	1.86 (1.61 to 2.11)	1.86 (1.61 to 2.11)	1.86 (1.61 to 2.11)	1.86 (1.61 to 2.11)	1.86 (1.61 to 2.11)	1.86 (1.61 to 2.11)	1.86 (1.61 to 2.11)	1.86 (1.61 to 2.11)
Pancreatic cancer	10 Pack Years	Both	Male	1.41 (1.24 to 1.59)	1.41 (1.24 to 1.59)	1.41 (1.24 to 1.59)	1.41 (1.24 to 1.59)	1.41 (1.24 to 1.59)	1.41 (1.24 to 1.59)	1.41 (1.24 to 1.59)	1.41 (1.24 to 1.59)	1.41 (1.24 to 1.59)	1.41 (1.24 to 1.59)	1.41 (1.24 to 1.59)	1.41 (1.24 to 1.59)	1.41 (1.24 to 1.59)	1.41 (1.24 to 1.59)
Pancreatic cancer	20 Pack Years	Both	Female	2.46 (2.03 to 2.91)	2.46 (2.03 to 2.91)	2.46 (2.03 to 2.91)	2.46 (2.03 to 2.91)	2.46 (2.03 to 2.91)	2.46 (2.03 to 2.91)	2.46 (2.03 to 2.91)	2.46 (2.03 to 2.91)	2.46 (2.03 to 2.91)	2.46 (2.03 to 2.91)	2.46 (2.03 to 2.91)	2.46 (2.03 to 2.91)	2.46 (2.03 to 2.91)	2.46 (2.03 to 2.91)
Pancreatic cancer	20 Pack Years	Both	Male	1.69 (1.41 to 1.98)	1.69 (1.41 to 1.98)	1.69 (1.41 to 1.98)	1.69 (1.41 to 1.98)	1.69 (1.41 to 1.98)	1.69 (1.41 to 1.98)	1.69 (1.41 to 1.98)	1.69 (1.41 to 1.98)	1.69 (1.41 to 1.98)	1.69 (1.41 to 1.98)	1.69 (1.41 to 1.98)	1.69 (1.41 to 1.98)	1.69 (1.41 to 1.98)	1.69 (1.41 to 1.98)
Pancreatic cancer	30 Pack Years	Both	Female	2.71 (2.18 to 3.27)	2.71 (2.18 to 3.27)	2.71 (2.18 to 3.27)	2.71 (2.18 to 3.27)	2.71 (2.18 to 3.27)	2.71 (2.18 to 3.27)	2.71 (2.18 to 3.27)	2.71 (2.18 to 3.27)	2.71 (2.18 to 3.27)	2.71 (2.18 to 3.27)	2.71 (2.18 to 3.27)	2.71 (2.18 to 3.27)	2.71 (2.18 to 3.27)	2.71 (2.18 to 3.27)
Pancreatic cancer	30 Pack Years	Both	Male	1.79 (1.50 to 2.11)	1.79 (1.50 to 2.11)	1.79 (1.50 to 2.11)	1.79 (1.50 to 2.11)	1.79 (1.50 to 2.11)	1.79 (1.50 to 2.11)	1.79 (1.50 to 2.11)	1.79 (1.50 to 2.11)	1.79 (1.50 to 2.11)	1.79 (1.50 to 2.11)	1.79 (1.50 to 2.11)	1.79 (1.50 to 2.11)	1.79 (1.50 to 2.11)	1.79 (1.50 to 2.11)
Pancreatic cancer	40 Pack Years	Both	Female	3.03 (2.36 to 3.77)	3.03 (2.36 to 3.77)	3.03 (2.36 to 3.77)	3.03 (2.36 to 3.77)	3.03 (2.36 to 3.77)	3.03 (2.36 to 3.77)	3.03 (2.36 to 3.77)	3.03 (2.36 to 3.77)	3.03 (2.36 to 3.77)	3.03 (2.36 to 3.77)	3.03 (2.36 to 3.77)	3.03 (2.36 to 3.77)	3.03 (2.36 to 3.77)	3.03 (2.36 to 3.77)
Pancreatic cancer	40 Pack Years	Both	Male	2.00 (1.67 to 2.34)	2.00 (1.67 to 2.34)	2.00 (1.67 to 2.34)	2.00 (1.67 to 2.34)	2.00 (1.67 to 2.34)	2.00 (1.67 to 2.34)	2.00 (1.67 to 2.34)	2.00 (1.67 to 2.34)	2.00 (1.67 to 2.34)	2.00 (1.67 to 2.34)	2.00 (1.67 to 2.34)	2.00 (1.67 to 2.34)	2.00 (1.67 to 2.34)	2.00 (1.67 to 2.34)
Pancreatic cancer	50 Pack Years	Both	Female	3.38 (2.46 to 4.42)	3.38 (2.46 to 4.42)	3.38 (2.46 to 4.42)	3.38 (2.46 to 4.42)	3.38 (2.46 to 4.42)	3.38 (2.46 to 4.42)	3.38 (2.46 to 4.42)	3.38 (2.46 to 4.42)	3.38 (2.46 to 4.42)	3.38 (2.46 to 4.42)	3.38 (2.46 to 4.42)	3.38 (2.46 to 4.42)	3.38 (2.46 to 4.42)	3.38 (2.46 to 4.42)
Pancreatic cancer	50 Pack Years	Both	Male	2.22 (1.81 to 2.61)	2.22 (1.81 to 2.61)	2.22 (1.81 to 2.61)	2.22 (1.81 to 2.61)	2.22 (1.81 to 2.61)	2.22 (1.81 to 2.61)	2.22 (1.81 to 2.61)	2.22 (1.81 to 2.61)	2.22 (1.81 to 2.61)	2.22 (1.81 to 2.61)	2.22 (1.81 to 2.61)	2.22 (1.81 to 2.61)	2.22 (1.81 to 2.61)	2.22 (1.81 to 2.61)
Pancreatic cancer	60 Pack Years	Both	Female	3.74 (2.36 to 5.38)	3.74 (2.36 to 5.38)	3.74 (2.36 to 5.38)	3.74 (2.36 to 5.38)	3.74 (2.36 to 5.38)	3.74 (2.36 to 5.38)	3.74 (2.36 to 5.38)	3.74 (2.36 to 5.38)	3.74 (2.36 to 5.38)	3.74 (2.36 to 5.38)	3.74 (2.36 to 5.38)	3.74 (2.36 to 5.38)	3.74 (2.36 to 5.38)	3.74 (2.36 to 5.38)
Pancreatic cancer	60 Pack Years	Both	Male	2.38 (1.84 to 2.97)	2.38 (1.84 to 2.97)	2.38 (1.84 to 2.97)	2.38 (1.84 to 2.97)	2.38 (1.84 to 2.97)	2.38 (1.84 to 2.97)	2.38 (1.84 to 2.97)	2.38 (1.84 to 2.97)	2.38 (1.84 to 2.97)	2.38 (1.84 to 2.97)	2.38 (1.84 to 2.97)	2.38 (1.84 to 2.97)	2.38 (1.84 to 2.97)	2.38 (1.84 to 2.97)
Pancreatic cancer	70 Pack Years	Both	Female	4.08 (2.20 to 6.75)	4.08 (2.20 to 6.75)	4.08 (2.20 to 6.75)	4.08 (2.20 to 6.75)	4.08 (2.20 to 6.75)	4.08 (2.20 to 6.75)	4.08 (2.20 to 6.75)	4.08 (2.20 to 6.75)	4.08 (2.20 to 6.75)	4.08 (2.20 to 6.75)	4.08 (2.20 to 6.75)	4.08 (2.20 to 6.75)	4.08 (2.20 to 6.75)	4.08 (2.20 to 6.75)
Pancreatic cancer	70 Pack Years	Both	Male	2.65 (1.87 to 3.60)	2.65 (1.87 to 3.60)	2.65 (1.87 to 3.60)	2.65 (1.87 to 3.60)	2.65 (1.87 to 3.60)	2.65 (1.87 to 3.60)	2.65 (1.87 to 3.60)	2.65 (1.87 to 3.60)	2.65 (1.87 to 3.60)	2.65 (1.87 to 3.60)	2.65 (1.87 to 3.60)	2.65 (1.87 to 3.60)	2.65 (1.87 to 3.60)	2.65 (1.87 to 3.60)

Supplementary File S3 Sociodemographic index (SDI)

The SDI is a composite indicator of social development status which strongly correlated with health outcomes, ranging from 0 to 1. It is the geometric mean of total fertility rate in those under 25 years old (TFU25), mean education for those age 15 years or older (EDU15+) and lag-distributed income per capita (LDI). The specific ranges of the three indicators are as follows:

Indicators	Lower Bound	Upper Bound
TFU25	0	3
EDU15+	0 years	17 years
LDI	250 USD	60,000 USD

The index scores underlying SDI are calculated as follows:

$$l_{cly} = \frac{(c_{ly} - c_{low})}{(c_{high} - c_{low})}$$

(l_{cly}: the index for covariate C, location l and year y)

The value of SDI in GBD 2019 contains 1062 national and subnational locations spanning the time period 1950 to 2019. The SDI values in 2019 by location are listed as follows.

Location	SDI in 2019
Afghanistan	0.343
Albania	0.681
Algeria	0.652
American Samoa	0.712
Andorra	0.894
Angola	0.47
Antigua and Barbuda	0.743
Argentina	0.708

Armenia	0.689
Australia	0.839
Austria	0.849
Azerbaijan	0.683
Bahamas	0.796
Bahrain	0.751
Bangladesh	0.483
Barbados	0.742
Belarus	0.745
Belgium	0.851
Belize	0.603
Benin	0.352
Bermuda	0.813
Bhutan	0.455
Bolivia (Plurinational State of)	0.566
Bosnia and Herzegovina	0.718
Botswana	0.634
Brazil	0.64
Brunei Darussalam	0.823
Bulgaria	0.764
Burkina Faso	0.257
Burundi	0.284
Cabo Verde	0.525
Cambodia	0.469
Cameroon	0.49
Canada	0.873
Central African Republic	0.274
Chad	0.238
Chile	0.759

China	0.686
Colombia	0.633
Comoros	0.455
Congo	0.568
Cook Islands	0.764
Costa Rica	0.68
Croatia	0.794
Cuba	0.668
Cyprus	0.841
Czechia	0.828
Cote d'Ivoire	0.408
Democratic People's Republic of Korea	0.558
Democratic Republic of the Congo	0.382
Denmark	0.89
Djibouti	0.459
Dominica	0.729
Dominican Republic	0.592
Ecuador	0.64
Egypt	0.658
El Salvador	0.573
Equatorial Guinea	0.685
Eritrea	0.396
Estonia	0.835
Eswatini	0.577
Ethiopia	0.343
Fiji	0.664
Finland	0.856
France	0.834

Gabon	0.656
Gambia	0.399
Georgia	0.702
Germany	0.898
Ghana	0.557
Greece	0.794
Greenland	0.761
Grenada	0.669
Guam	0.813
Guatemala	0.526
Guinea	0.325
Guinea-Bissau	0.355
Guyana	0.618
Haiti	0.432
Honduras	0.496
Hungary	0.791
Iceland	0.869
India	0.566
Indonesia	0.66
Iran (Islamic Republic of)	0.67
Iraq	0.671
Ireland	0.867
Israel	0.803
Italy	0.801
Jamaica	0.684
Japan	0.87
Jordan	0.731
Kazakhstan	0.723
Kenya	0.508

Kiribati	0.527
Kuwait	0.851
Kyrgyzstan	0.596
Lao People's Democratic Republic	0.49
Latvia	0.82
Lebanon	0.708
Lesotho	0.507
Liberia	0.37
Libya	0.709
Lithuania	0.843
Luxembourg	0.895
Madagascar	0.396
Malawi	0.384
Malaysia	0.737
Maldives	0.562
Mali	0.263
Malta	0.801
Marshall Islands	0.544
Mauritania	0.496
Mauritius	0.705
Mexico	0.649
Micronesia (Federated States of)	0.58
Monaco	0.902
Mongolia	0.606
Montenegro	0.791
Morocco	0.548
Mozambique	0.307
Myanmar	0.521
Namibia	0.612

Nauru	0.618
Nepal	0.422
Netherlands	0.883
New Zealand	0.84
Nicaragua	0.517
Niger	0.162
Nigeria	0.515
Niue	0.711
North Macedonia	0.744
Northern Mariana Islands	0.771
Norway	0.913
Oman	0.783
Pakistan	0.449
Palau	0.738
Palestine	0.588
Panama	0.686
Papua New Guinea	0.394
Paraguay	0.638
Peru	0.648
Philippines	0.623
Poland	0.802
Portugal	0.743
Puerto Rico	0.814
Qatar	0.83
Republic of Korea	0.878
Republic of Moldova	0.696
Romania	0.76
Russian Federation	0.805
Rwanda	0.429

Saint Kitts and Nevis	0.746
Saint Lucia	0.67
Saint Vincent and the Grenadines	0.627
Samoa	0.641
San Marino	0.884
Sao Tome and Principe	0.502
Saudi Arabia	0.805
Senegal	0.389
Serbia	0.767
Seychelles	0.724
Sierra Leone	0.347
Singapore	0.861
Slovakia	0.812
Slovenia	0.84
Solomon Islands	0.407
Somalia	0.081
South Africa	0.678
South Sudan	0.363
Spain	0.767
Sri Lanka	0.69
Sudan	0.515
Suriname	0.636
Sweden	0.872
Switzerland	0.929
Syrian Arab Republic	0.619
Taiwan (Province of China)	0.868
Tajikistan	0.539
Thailand	0.687
Timor-Leste	0.514

Togo	0.417
Tokelau	0.626
Tonga	0.636
Trinidad and Tobago	0.757
Tunisia	0.672
Turkey	0.748
Turkmenistan	0.67
Tuvalu	0.589
Uganda	0.404
Ukraine	0.736
United Arab Emirates	0.88
United Kingdom	0.847
United Republic of Tanzania	0.423
United States of America	0.859
United States Virgin Islands	0.799
Uruguay	0.697
Uzbekistan	0.631
Vanuatu	0.485
Venezuela (Bolivarian Republic of)	0.607
Viet Nam	0.617
Yemen	0.412
Zambia	0.505
Zimbabwe	0.476

Supplementary File S4 Joinpoint regression model

1. Overview

The Joinpoint regression model, developed by the National Cancer Institute, has been widely used to study trends in tumor morbidity and mortality. Joinpoint regression models are linear ($y = xb$) or log-linear ($\ln[y] = xb$). Grid search method is the default modeling method for Joinpoint. Monte Carlo permutation test is the default model selection method for Joinpoint software. The annual percentage change (APC) was used to estimate the rate of change in a given time period. The average annual percent change (AAPC), which provides a summary measure of the APCs over a period of time where the trend is not constant, was used to assess the trends in the incidence and mortality data of cancer disease.

2. Software

The Joinpoint software can be downloaded in National Cancer Institute (https://surveillance.cancer.gov/joinpoint/download)

3. Index calculation

The APC were calculated based on the following formulas:

$$APC = \left[\frac{y_{x_1} - y_x}{y_x}\right] \times 100 = (e^{\beta 1} - 1) \times 100$$

The 95% confidence intervals can be calculated as follows:

$$APC_l = 100(e^{\beta_1 - s \times t_d^{-1}(1-0.05/2)} - 1)$$

$$APC_u = 100(e^{\beta_1 + s \times t_d^{-1}(1-0.05/2)} - 1)$$

(β_1: regression coefficient; s: standard error of β_1; d: degree of freedom)

The AAPC is used to comprehensively evaluate global mean trends covering multiple intervals. The AAPC were calculated based on the following formulas:

$$AAPC = (exp\left(\frac{\sum w_i \beta_i}{\Sigma w_i}\right) - 1) \times 100$$

(w_i: the interval span width of each piecewise function; β_i: regression coefficients corresponding to each interval)

The 95% confidence intervals can be calculated as follows:

$$AAPC_l = \exp\left\{\ln\left[\left(\left(\frac{AAPC}{100}\right)+1\right) - Z_{1-\frac{0.05}{2}}\sqrt{\sum \widetilde{w}_i^2 \widetilde{\sigma}_i^2}\right] - 1\right\}$$

$$AAPC_u = \exp\left\{\ln\left[\left(\left(\frac{AAPC}{100}\right)+1\right) + Z_{1-\frac{0.05}{2}}\sqrt{\sum \widetilde{w}_i^2 \widetilde{\sigma}_i^2}\right] - 1\right\}$$

(w_i: the interval span width of each piecewise function; $\widetilde{w_i} = w_i/\sum w_i$; $\widetilde{\sigma_i^2}$ is the variance of β_1)

Figure S1 Age-specific counts of deaths (A) and DALYs (B) of pancreatic cancer attributable to smoking by sex, 2019.

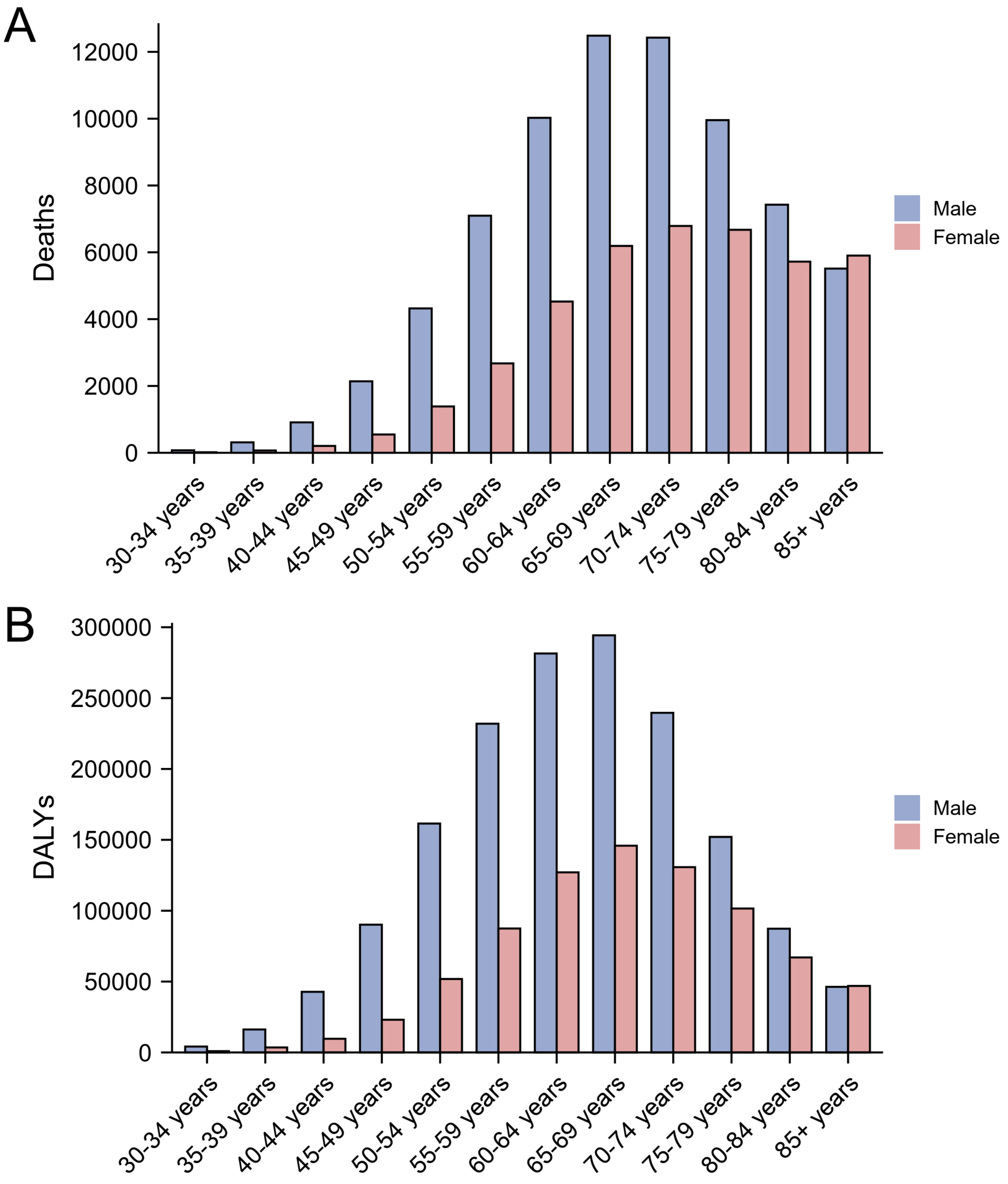

Figure S2 The trend in the proportion of deaths and DALYs attributable to smoking from 1990 to 2019. (A) Deaths; (B) DALYs.

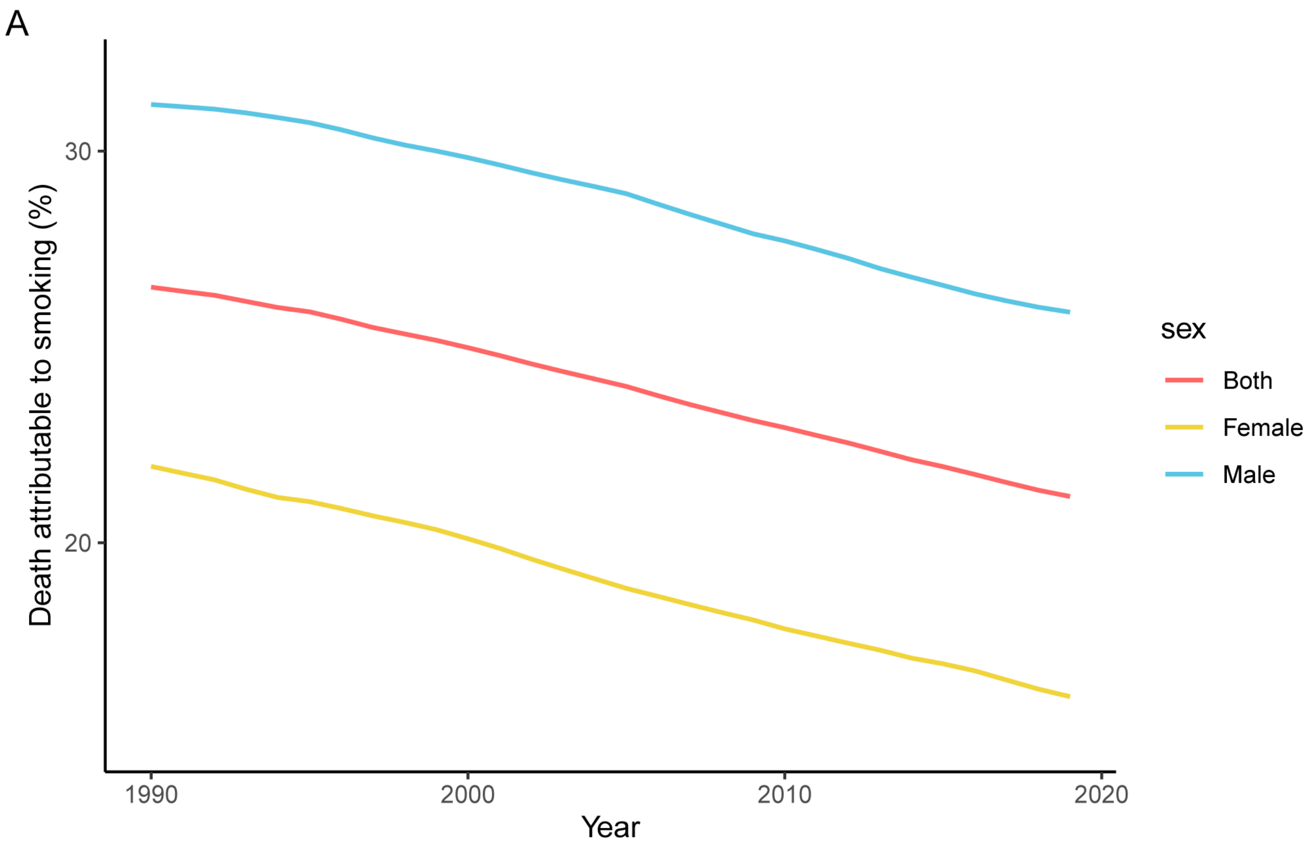

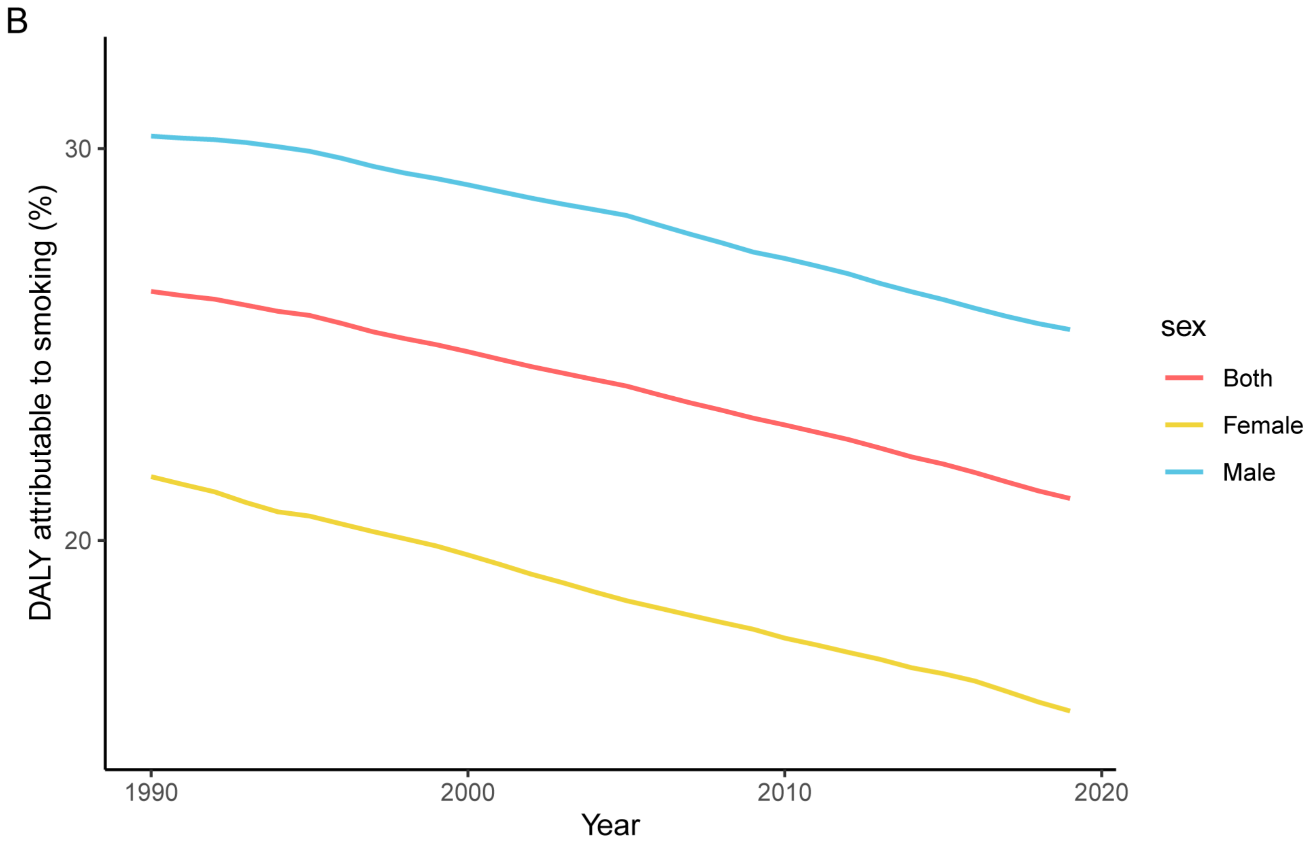

Figure S3 The proportion of age-specific deaths attributable to smoking for (A) males and (B) females in 2019.

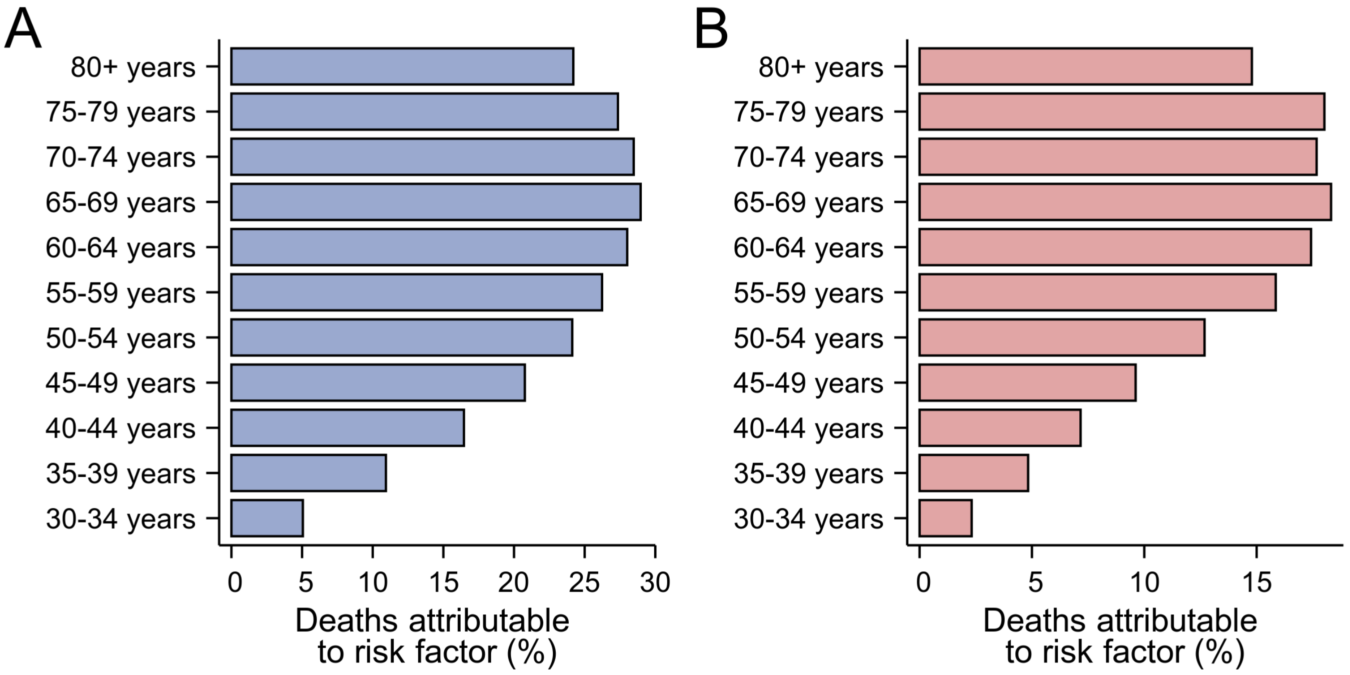

Figure S4 The change trend in the age-standardized DALY rate of pancreatic cancer attributable to smoking in four continents, 1990 to 2019. (A) Africa; (B) America; (C) Asia; (D) Europe.

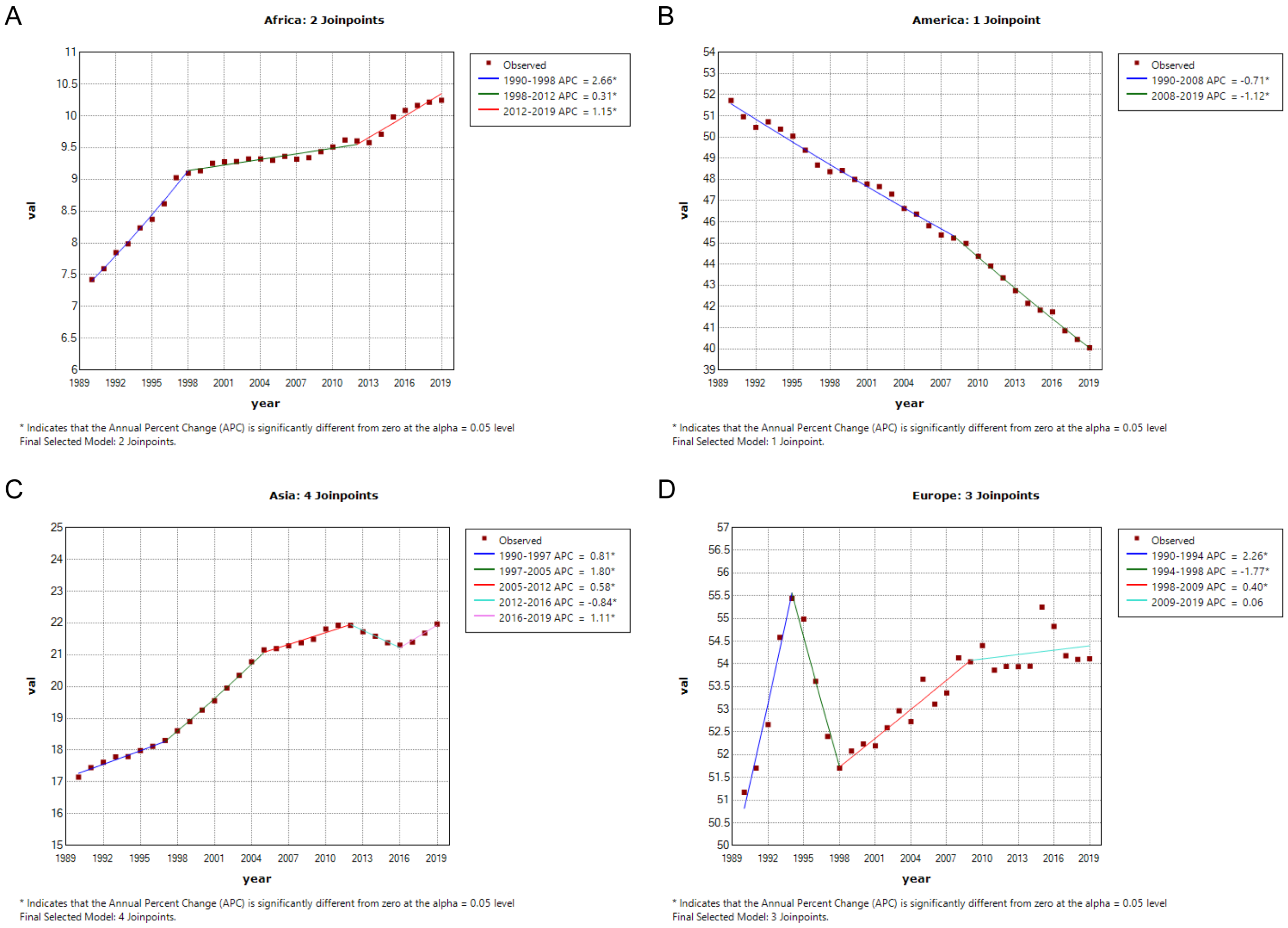

Figure S5 Cluster plot of countries/territories with the same AAPC.

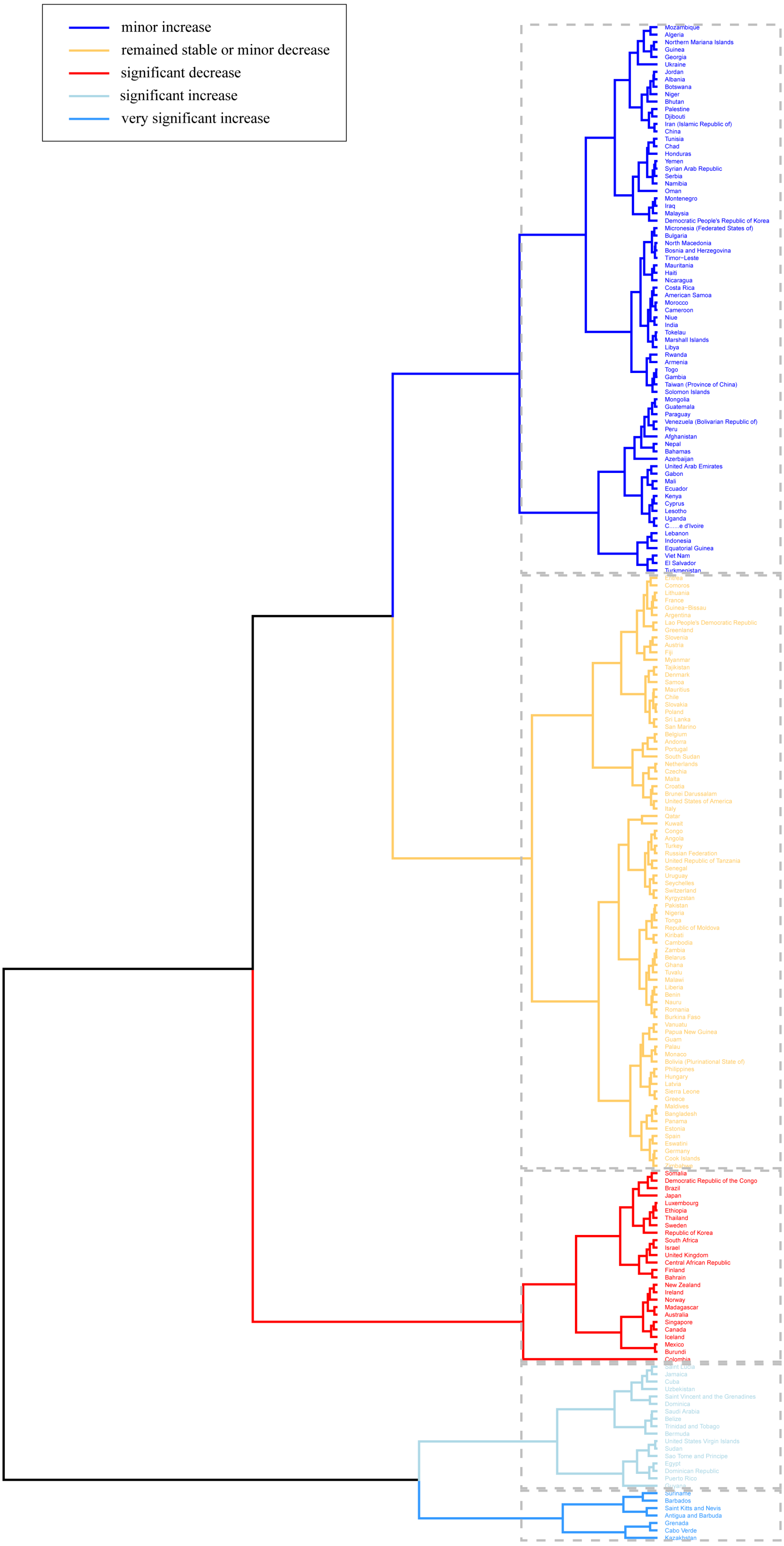

Figure S6 The correlation between AAPCs (1990 to 2019) and ASRs (in 2019), and HDI (in 2019).

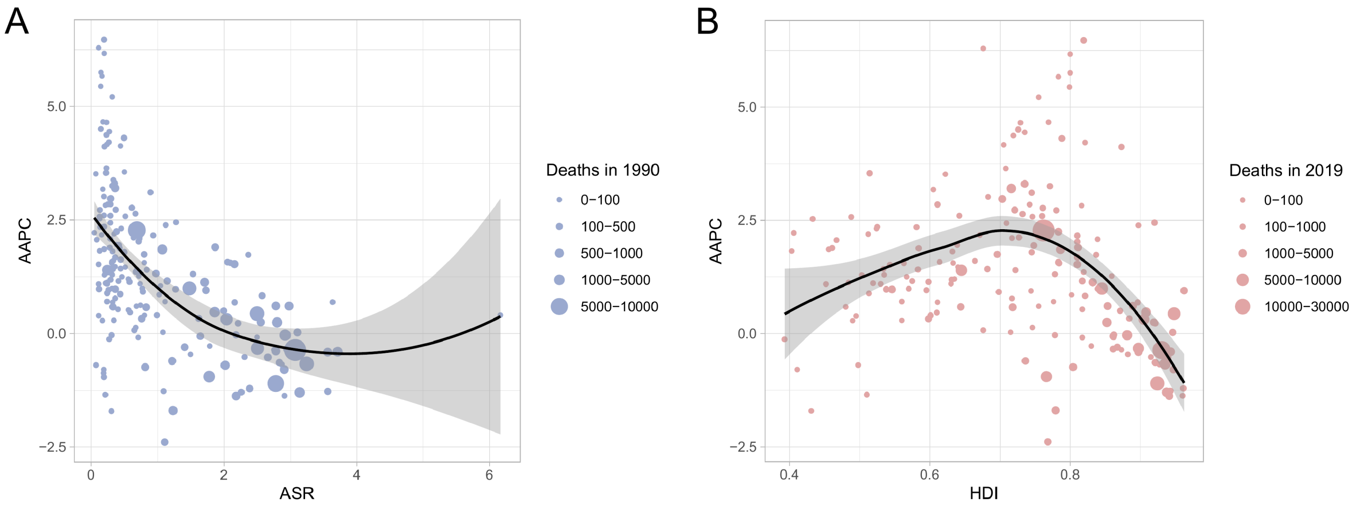

Figure S7 The change trend in ASDR of pancreatic cancer attributable to smoking in five SDI quintiles, 1990 to 2019. (A) High SDI; (B) high-middle SDI; (C) middle SDI; (D) low-middle SDI; (E) low SDI.

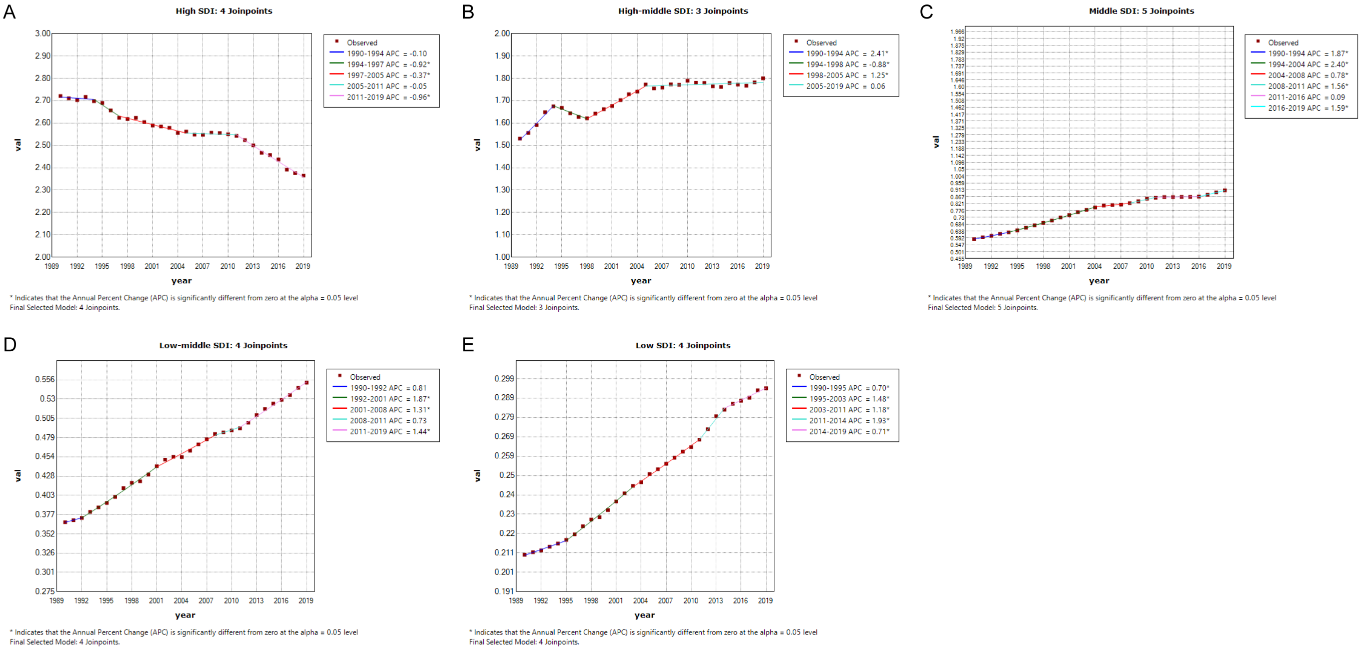

Figure S8 The change trend in the age-standardized DALY rate of pancreatic cancer attributable to smoking in five SDI quintiles, 1990 to 2019. (A) High SDI; (B) high-middle SDI; (C) middle SDI; (D) low-middle SDI; (E) low SDI.

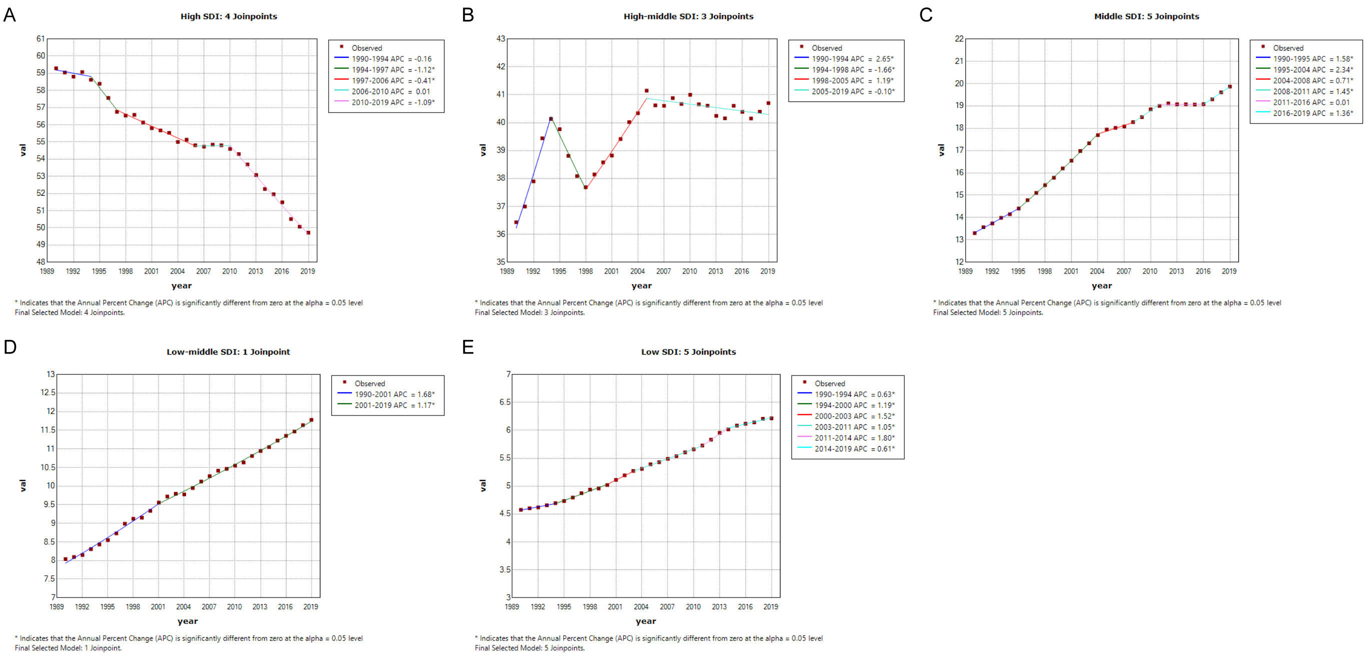

Figure S9 The trend in the age-standardized DALY rate of smoking-related pancreatic cancer across 21 GBD regions by SDI, from 1990 to 2019

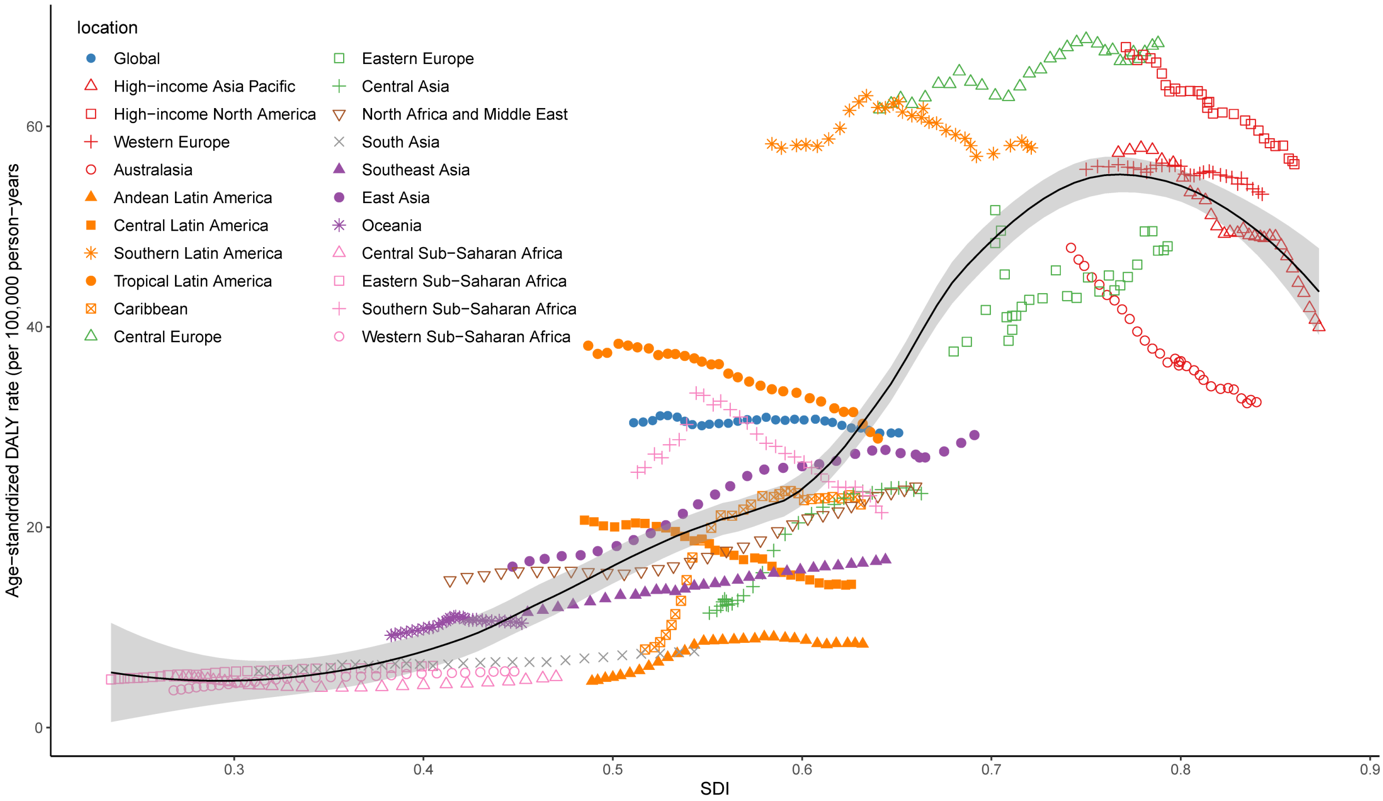

Figure S10 The relationship between the age-standardized DALY rate of smoking -related pancreatic cancer and SDI among all countries/territories in 2019

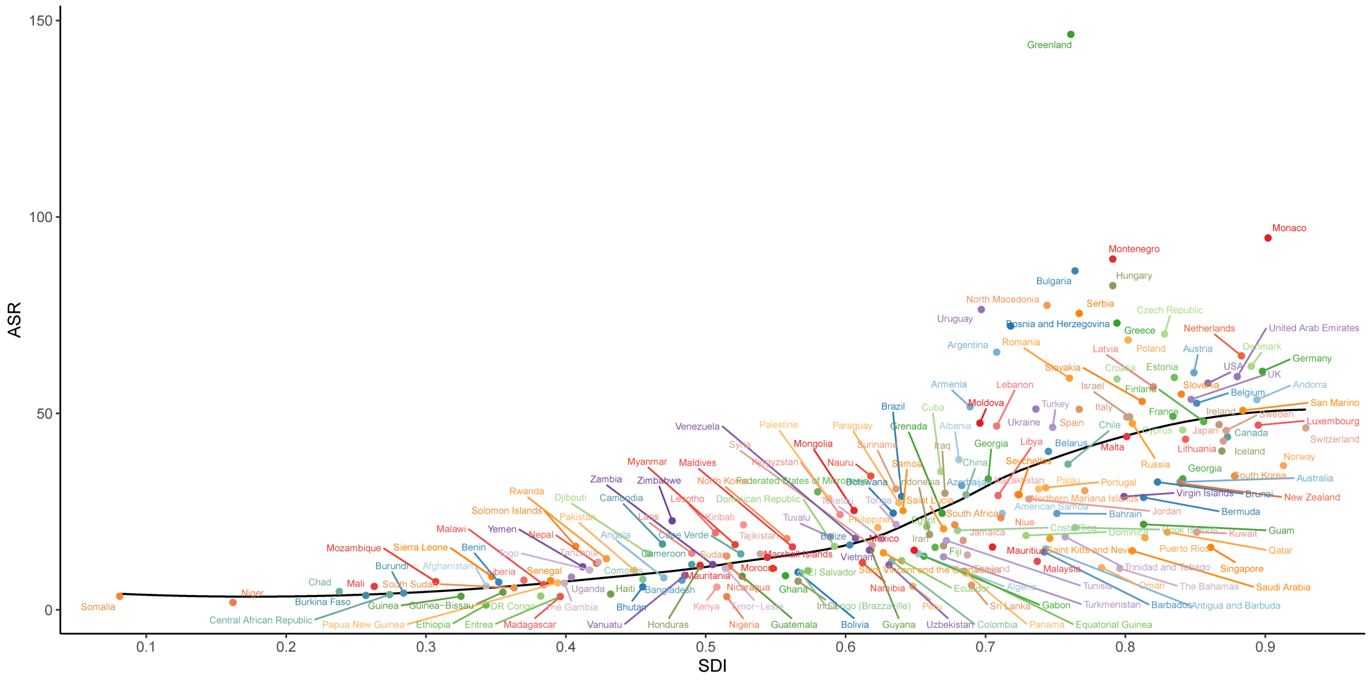

Table S1 DALYs and age-standardized DALY rate of smoking-attributable pancreatic cancer among all regions, 1990 to and 2019

	DALYs in 1990	Age-standardized DALY rate in 1990	DALYs in 2019	Age-standardized DALY rate in 2019
Global	1217116 (1081399 to 1346126)	30.43 (27.14 to 33.67)	2443356 (2108770 to 2774321)	29.42 (25.43 to 33.4)
Different SDI				
High SDI	612508 (546317 to 673234)	59.29 (52.97 to 65.25)	894161 (787252 to 1013373)	49.72 (44.02 to 56.17)
High-middle SDI	402273 (352461 to 448864)	36.43 (31.98 to 40.58)	841567 (722765 to 966481)	40.7 (34.94 to 46.79)
Middle SDI	142204 (115979 to 168730)	13.31 (11.05 to 15.67)	512528 (414801 to 627344)	19.87 (16.23 to 24.1)
Low-middle SDI	48822 (36395 to 63207)	8.04 (6.11 to 10.21)	162323 (130842 to 193902)	11.78 (9.52 to 14)
Low SDI	10898 (7297 to 14977)	4.58 (3.07 to 6.22)	31700 (23535 to 40192)	6.21 (4.69 to 7.77)
GBD Region				
Andean Latin America	941 (689 to 1239)	4.63 (3.44 to 6.05)	4643 (3296 to 6296)	8.34 (5.95 to 11.29)
Australasia	11253 (9920 to 12555)	47.88 (42.37 to 53.38)	15147 (12864 to 17664)	32.48 (27.49 to 37.96)
Caribbean	2033 (1734 to 2316)	7.8 (6.67 to 8.9)	11582 (9120 to 14243)	22.27 (17.57 to 27.42)
Central Asia	5616 (4716 to 6577)	11.44 (9.58 to 13.39)	18648 (15660 to 21926)	23.36 (19.71 to 27.25)
Central Europe	93219 (82657 to 103367)	61.7 (54.79 to 68.33)	140156 (118466 to 165830)	68.31 (57.33 to 80.85)
Central Latin America	17242 (13914 to 20325)	20.71 (16.96 to 24.14)	33739 (25379 to 43626)	14.3 (10.76 to 18.44)

Central Sub-Saharan Africa	1215 (738 to 1838)	5.01 (3.1 to 7.44)	2850 (1790 to 4111)	5.05 (3.23 to 7.16)
East Asia	148451 (114905 to 186875)	16.06 (12.64 to 19.95)	631986 (493276 to 800825)	29.19 (22.87 to 36.85)
Eastern Europe	107757 (91652 to 124901)	37.54 (31.87 to 43.45)	160773 (134729 to 190986)	48.03 (40.02 to 56.9)
Eastern Sub-Saharan Africa	3604 (2394 to 4986)	4.81 (3.24 to 6.61)	9847 (6583 to 13420)	6.16 (4.23 to 8.33)
High-income Asia Pacific	118441 (104591 to 132253)	57.36 (50.73 to 64.1)	163620 (141633 to 185928)	39.98 (34.66 to 45.14)
High-income North America	232084 (201453 to 261675)	67.9 (59.32 to 76.08)	346554 (297828 to 397768)	56.23 (48.87 to 64.15)
North Africa and Middle East	26734 (20979 to 34137)	14.71 (11.65 to 18.64)	108398 (88697 to 131758)	24.05 (19.9 to 28.97)
Oceania	287 (208 to 381)	9.21 (6.75 to 12.08)	762 (536 to 1034)	10.42 (7.62 to 13.91)
South Asia	31514 (21264 to 43330)	5.63 (3.86 to 7.61)	106416 (78606 to 134790)	7.61 (5.72 to 9.6)
Southeast Asia	30366 (23996 to 36621)	11.5 (9.33 to 13.68)	104514 (76540 to 140948)	16.75 (12.44 to 22.32)
Southern Latin America	27441 (23476 to 31223)	58.26 (49.8 to 66.27)	47727 (40695 to 54687)	57.86 (49.4 to 66.39)
Southern Sub-Saharan Africa	7158 (5580 to 9130)	25.49 (19.87 to 32.88)	12295 (9574 to 15070)	21.45 (16.96 to 26.11)
Tropical Latin America	35407 (30995 to 39704)	38.12 (33.45 to 42.94)	70893 (59406 to 82156)	28.85 (24.18 to 33.54)
Western Europe	313009 (278112 to 346402)	55.71 (49.45 to 61.6)	442190 (389766 to 503521)	53.23 (46.71 to 60.39)
Western Sub-Saharan Africa	3344 (2293 to 4671)	3.72 (2.59 to 5.12)	10616 (7260 to 14437)	5.61 (3.91 to 7.53)

DALY: disability-adjusted life-year; SDI: socio-demographic index.

Table S2 Death and its change trends in smoking-related pancreatic cancer from 1990 to 2019 among all countries/territories

Country/region	Deaths in 1990	ASDR in 1990	Deaths in 2019	ASDR in 2019	AAPC and 95% CI
Afghanistan	8 (4 to 17)	0.13 (0.06 to 0.24)	29 (18 to 49)	0.26 (0.16 to 0.41)	2.574 (2.325 to 2.824)
Albania	18 (16 to 22)	0.93 (0.79 to 1.11)	78 (56 to 106)	1.78 (1.27 to 2.41)	2.162 (1.745 to 2.581)
Algeria	43 (33 to 56)	0.42 (0.32 to 0.53)	218 (164 to 279)	0.73 (0.55 to 0.94)	1.946 (1.772 to 2.119)
American Samoa	0 (0 to 0)	0.73 (0.55 to 0.93)	0 (0 to 1)	1.05 (0.81 to 1.35)	1.349 (0.638 to 2.065)
Andorra	1 (1 to 2)	2.75 (1.99 to 3.88)	3 (3 to 5)	2.47 (1.82 to 3.28)	–0.356 (–0.481 to –0.231)
Angola	10 (6 to 15)	0.26 (0.17 to 0.39)	36 (25 to 50)	0.35 (0.25 to 0.47)	0.96 (0.689 to 1.231)
Antigua and Barbuda	0 (0 to 0)	0.15 (0.11 to 0.19)	1 (1 to 1)	0.75 (0.55 to 0.96)	5.754 (4.837 to 6.68)
Argentina	912 (765 to 1055)	2.8 (2.36 to 3.24)	1630 (1377 to 1897)	2.99 (2.53 to 3.48)	0.247 (–0.046 to 0.541)
Armenia	41 (33 to 52)	1.53 (1.24 to 1.91)	93 (73 to 115)	2.21 (1.76 to 2.73)	1.31 (0.938 to 1.683)
Australia	432 (376 to 487)	2.18 (1.89 to 2.46)	615 (514 to 734)	1.47 (1.24 to 1.74)	–1.374 (–1.565 to –1.182)
Austria	307 (264 to 354)	2.55 (2.21 to 2.92)	493 (423 to 571)	2.76 (2.39 to 3.18)	0.251 (0.053 to 0.448)
Azerbaijan	31 (24 to 40)	0.61 (0.47 to 0.8)	121 (92 to 158)	1.36 (1.05 to 1.74)	2.771 (2.483 to 3.06)
Bahamas	0 (0 to 0)	0.22 (0.17 to 0.27)	2 (1 to 2)	0.47 (0.35 to 0.62)	2.825 (2.509 to 3.143)
Bahrain	2 (2 to 3)	1.5 (1.15 to 1.9)	9 (7 to 12)	1.33 (1 to 1.76)	–0.458 (–1.056 to 0.144)
Bangladesh	138 (77 to 207)	0.33 (0.18 to 0.48)	458 (258 to 720)	0.37 (0.21 to 0.59)	0.589 (0.228 to 0.951)
Barbados	0 (0 to 1)	0.14 (0.12 to 0.18)	3 (3 to 4)	0.67 (0.51 to 0.87)	5.441 (4.879 to 6.005)
Belarus	151 (129 to 175)	1.15 (0.98 to 1.33)	248 (185 to 338)	1.56 (1.17 to 2.12)	1.159 (0.636 to 1.685)
Belgium	440 (379 to 501)	2.78 (2.4 to 3.15)	572 (489 to 672)	2.48 (2.14 to 2.89)	–0.385 (–0.659 to –0.111)
Belize	0 (0 to 0)	0.24 (0.19 to 0.29)	2 (1 to 2)	0.74 (0.57 to 0.96)	4.164 (3.582 to 4.749)
Benin	5 (3 to 6)	0.24 (0.16 to 0.33)	14 (9 to 20)	0.33 (0.21 to 0.46)	1.095 (0.81 to 1.382)
Bermuda	0 (0 to 0)	0.44 (0.34 to 0.56)	2 (1 to 3)	1.42 (1.06 to 1.89)	4.131 (3.598 to 4.667)
Bhutan	0 (0 to 1)	0.16 (0.07 to 0.26)	2 (1 to 2)	0.3 (0.16 to 0.45)	2.174 (2.071 to 2.277)
Bolivia (Plurinational State of)	11 (7 to 15)	0.36 (0.23 to 0.5)	38 (23 to 59)	0.45 (0.27 to 0.68)	0.776 (0.575 to 0.977)
Bosnia and Herzegovina	81 (69 to 94)	2.05 (1.75 to 2.39)	195 (149 to 253)	3.21 (2.45 to 4.16)	1.573 (1.119 to 2.03)

Botswana	3 (2 to 4)	0.64 (0.46 to 0.85)	14 (9 to 19)	1.17 (0.82 to 1.63)	2.11 (1.898 to 2.322)
Brazil	1468 (1286 to 1654)	1.78 (1.55 to 2.01)	3138 (2648 to 3652)	1.35 (1.14 to 1.57)	–0.948 (–1.108 to –0.788)
Brunei Darussalam	1 (1 to 2)	1.78 (1.41 to 2.24)	4 (3 to 5)	1.63 (1.26 to 2.03)	–0.273 (–0.696 to 0.153)
Bulgaria	280 (246 to 315)	2.16 (1.9 to 2.43)	451 (343 to 582)	3.25 (2.46 to 4.21)	1.528 (0.987 to 2.072)
Burkina Faso	5 (3 to 8)	0.11 (0.06 to 0.18)	13 (7 to 21)	0.15 (0.09 to 0.24)	1.092 (0.962 to 1.223)
Burundi	7 (4 to 10)	0.3 (0.2 to 0.43)	8 (5 to 12)	0.18 (0.11 to 0.28)	–1.707 (–1.936 to –1.477)
Cabo Verde	0 (0 to 0)	0.11 (0.08 to 0.14)	3 (2 to 4)	0.65 (0.44 to 0.9)	6.292 (5.641 to 6.947)
Cambodia	23 (15 to 33)	0.58 (0.38 to 0.81)	86 (65 to 106)	0.81 (0.62 to 0.97)	1.14 (0.984 to 1.296)
Cameroon	14 (9 to 21)	0.33 (0.21 to 0.48)	55 (33 to 84)	0.49 (0.3 to 0.73)	1.417 (1.317 to 1.516)
Canada	1026 (895 to 1166)	3.14 (2.73 to 3.56)	1517 (1259 to 1826)	2.14 (1.78 to 2.56)	–1.295 (–1.503 to –1.086)
Central African Republic	2 (1 to 4)	0.19 (0.11 to 0.31)	3 (2 to 5)	0.15 (0.09 to 0.24)	–0.792 (–0.963 to –0.622)
Chad	3 (2 to 5)	0.13 (0.08 to 0.18)	11 (7 to 16)	0.22 (0.14 to 0.31)	1.821 (1.678 to 1.964)
Chile	164 (136 to 193)	1.64 (1.36 to 1.94)	390 (319 to 468)	1.61 (1.32 to 1.93)	–0.058 (–0.38 to 0.265)
China	5529 (4381 to 6837)	0.69 (0.56 to 0.84)	26552 (20856 to 33174)	1.34 (1.05 to 1.65)	2.277 (2.021 to 2.534)
Colombia	185 (149 to 218)	1.11 (0.89 to 1.31)	296 (210 to 409)	0.56 (0.4 to 0.78)	–2.389 (–3.008 to –1.767)
Comoros	1 (0 to 1)	0.34 (0.2 to 0.48)	2 (1 to 2)	0.37 (0.23 to 0.52)	0.291 (–0.128 to 0.712)
Congo	3 (2 to 5)	0.31 (0.2 to 0.47)	10 (6 to 16)	0.41 (0.26 to 0.64)	0.991 (0.846 to 1.137)
Cook Islands	0 (0 to 0)	0.8 (0.59 to 1.06)	0 (0 to 0)	0.91 (0.68 to 1.19)	0.401 (0.167 to 0.636)
Costa Rica	11 (9 to 13)	0.67 (0.55 to 0.8)	50 (36 to 68)	0.99 (0.72 to 1.34)	1.359 (0.58 to 2.144)
Croatia	189 (164 to 215)	2.95 (2.56 to 3.37)	233 (178 to 302)	2.63 (2 to 3.41)	–0.34 (–1.069 to 0.394)
Cuba	51 (44 to 59)	0.5 (0.43 to 0.57)	305 (238 to 379)	1.59 (1.24 to 1.98)	4.308 (3.716 to 4.903)
Cyprus	9 (7 to 11)	1.12 (0.94 to 1.34)	43 (35 to 52)	2.18 (1.79 to 2.62)	2.389 (2.162 to 2.617)
Czechia	496 (432 to 565)	3.56 (3.12 to 4.04)	692 (531 to 872)	3.2 (2.45 to 4.04)	–0.408 (–0.992 to 0.179)
Côte d'Ivoire	10 (7 to 14)	0.26 (0.18 to 0.36)	50 (34 to 69)	0.52 (0.37 to 0.69)	2.4 (2.168 to 2.634)
Democratic People's Republic of Korea	73 (51 to 104)	0.44 (0.31 to 0.61)	231 (167 to 300)	0.71 (0.52 to 0.92)	1.629 (1.55 to 1.708)
Democratic Republic of the Congo	29 (16 to 44)	0.19 (0.11 to 0.3)	49 (28 to 75)	0.15 (0.08 to 0.22)	–0.958 (–1.125 to –0.791)

Denmark	260 (229 to 290)	3.1 (2.75 to 3.46)	377 (318 to 443)	3.13 (2.65 to 3.66)	0.026 (–0.162 to 0.213)
Djibouti	0 (0 to 1)	0.34 (0.22 to 0.52)	3 (2 to 5)	0.65 (0.4 to 0.94)	2.194 (1.984 to 2.404)
Dominica	0 (0 to 0)	0.23 (0.18 to 0.29)	1 (1 to 1)	0.86 (0.63 to 1.17)	4.651 (4.364 to 4.938)
Dominican Republic	11 (8 to 13)	0.31 (0.25 to 0.39)	70 (46 to 98)	0.79 (0.53 to 1.1)	3.254 (2.983 to 3.525)
Ecuador	14 (11 to 17)	0.29 (0.23 to 0.35)	90 (65 to 121)	0.62 (0.45 to 0.83)	2.595 (2.198 to 2.993)
Egypt	100 (81 to 122)	0.35 (0.29 to 0.42)	548 (368 to 818)	0.88 (0.6 to 1.29)	3.304 (2.885 to 3.725)
El Salvador	6 (4 to 7)	0.19 (0.15 to 0.24)	26 (18 to 37)	0.44 (0.3 to 0.63)	3.016 (2.391 to 3.644)
Equatorial Guinea	0 (0 to 1)	0.18 (0.08 to 0.33)	2 (1 to 3)	0.43 (0.23 to 0.76)	3.18 (2.974 to 3.385)
Eritrea	1 (1 to 2)	0.11 (0.07 to 0.17)	4 (2 to 6)	0.13 (0.07 to 0.2)	0.392 (0.262 to 0.523)
Estonia	45 (38 to 52)	2.16 (1.83 to 2.48)	68 (51 to 87)	2.57 (1.94 to 3.33)	0.688 (–0.2 to 1.583)
Eswatini	1 (1 to 2)	0.56 (0.37 to 0.82)	3 (2 to 5)	0.66 (0.41 to 0.98)	0.521 (0.252 to 0.792)
Ethiopia	13 (5 to 27)	0.07 (0.03 to 0.14)	22 (11 to 38)	0.06 (0.03 to 0.1)	–0.694 (–0.842 to –0.545)
Fiji	2 (2 to 3)	0.67 (0.5 to 0.86)	5 (3 to 7)	0.7 (0.5 to 0.98)	0.133 (0.028 to 0.238)
Finland	193 (162 to 228)	2.66 (2.25 to 3.11)	282 (235 to 339)	2.26 (1.89 to 2.7)	–0.524 (–0.644 to –0.405)
France	1691 (1457 to 1930)	2.04 (1.75 to 2.32)	3105 (2587 to 3683)	2.26 (1.89 to 2.66)	0.315 (0.153 to 0.477)
Gabon	2 (1 to 3)	0.29 (0.18 to 0.47)	6 (3 to 10)	0.58 (0.34 to 0.94)	2.438 (2.246 to 2.63)
Gambia	1 (0 to 1)	0.2 (0.14 to 0.28)	3 (2 to 4)	0.29 (0.19 to 0.44)	1.293 (0.863 to 1.724)
Georgia	46 (36 to 59)	0.71 (0.57 to 0.92)	74 (59 to 92)	1.27 (1 to 1.57)	2.029 (0.881 to 3.19)
Germany	3234 (2781 to 3738)	2.5 (2.17 to 2.87)	5463 (4624 to 6415)	2.83 (2.44 to 3.28)	0.44 (0.184 to 0.697)
Ghana	16 (11 to 22)	0.29 (0.2 to 0.4)	58 (36 to 89)	0.41 (0.25 to 0.62)	1.188 (0.889 to 1.488)
Greece	433 (378 to 491)	2.78 (2.44 to 3.14)	779 (669 to 904)	3.31 (2.89 to 3.81)	0.61 (0.458 to 0.762)
Greenland	2 (2 to 2)	6.16 (5.11 to 7.38)	4 (4 to 6)	6.68 (5.24 to 8.31)	0.405 (–0.098 to 0.911)
Grenada	0 (0 to 0)	0.2 (0.16 to 0.25)	1 (1 to 2)	1.09 (0.83 to 1.38)	6.169 (5.555 to 6.786)
Guam	1 (0 to 1)	0.74 (0.55 to 0.95)	2 (1 to 2)	0.9 (0.68 to 1.18)	0.625 (0.149 to 1.103)
Guatemala	7 (4 to 9)	0.19 (0.14 to 0.25)	43 (29 to 60)	0.41 (0.28 to 0.57)	2.602 (2.015 to 3.193)
Guinea	3 (2 to 4)	0.08 (0.05 to 0.12)	8 (5 to 11)	0.15 (0.1 to 0.2)	2.065 (1.939 to 2.192)

Guinea-Bissau	1 (0 to 1)	0.17 (0.09 to 0.28)	1 (1 to 2)	0.19 (0.11 to 0.28)	0.286 (0.215 to 0.357)
Guyana	1 (1 to 1)	0.23 (0.18 to 0.29)	4 (3 to 5)	0.64 (0.45 to 0.88)	3.642 (3.235 to 4.05)
Haiti	4 (2 to 5)	0.11 (0.07 to 0.16)	11 (7 to 17)	0.17 (0.11 to 0.26)	1.522 (1.423 to 1.621)
Honduras	6 (4 to 8)	0.32 (0.23 to 0.41)	30 (16 to 47)	0.52 (0.28 to 0.8)	1.81 (1.125 to 2.499)
Hungary	445 (387 to 503)	2.98 (2.6 to 3.37)	661 (524 to 830)	3.43 (2.73 to 4.32)	0.607 (0.184 to 1.031)
Iceland	8 (7 to 10)	2.91 (2.43 to 3.42)	11 (9 to 14)	1.97 (1.61 to 2.39)	–1.369 (–1.695 to –1.043)
India	937 (637 to 1283)	0.24 (0.17 to 0.33)	3744 (2844 to 4701)	0.36 (0.27 to 0.44)	1.398 (0.894 to 1.904)
Indonesia	325 (244 to 411)	0.36 (0.27 to 0.45)	1747 (1119 to 2644)	0.9 (0.57 to 1.35)	3.204 (3.114 to 3.293)
Iran (Islamic Republic of)	96 (71 to 127)	0.38 (0.28 to 0.5)	507 (422 to 595)	0.73 (0.61 to 0.86)	2.247 (1.935 to 2.56)
Iraq	58 (38 to 81)	0.8 (0.52 to 1.11)	280 (206 to 363)	1.34 (1 to 1.72)	1.746 (1.552 to 1.941)
Ireland	148 (130 to 166)	3.56 (3.12 to 4)	188 (156 to 222)	2.45 (2.05 to 2.89)	–1.272 (–1.484 to –1.06)
Israel	139 (120 to 161)	2.83 (2.46 to 3.26)	279 (235 to 333)	2.37 (2 to 2.82)	–0.638 (–0.964 to –0.31)
Italy	2268 (1960 to 2567)	2.5 (2.17 to 2.83)	3378 (2864 to 3910)	2.32 (1.99 to 2.66)	–0.324 (–0.429 to –0.219)
Jamaica	4 (3 to 5)	0.23 (0.19 to 0.27)	23 (17 to 31)	0.78 (0.59 to 1.06)	4.373 (2.659 to 6.116)
Japan	4759 (4200 to 5379)	2.78 (2.45 to 3.14)	7434 (6196 to 8628)	2.04 (1.75 to 2.34)	–1.099 (–1.218 to –0.979)
Jordan	9 (7 to 11)	0.73 (0.58 to 0.92)	77 (59 to 98)	1.33 (1.02 to 1.69)	2.124 (1.67 to 2.58)
Kazakhstan	24 (20 to 29)	0.2 (0.16 to 0.24)	206 (163 to 251)	1.15 (0.92 to 1.4)	6.469 (5.793 to 7.149)
Kenya	10 (6 to 16)	0.14 (0.08 to 0.21)	52 (36 to 73)	0.27 (0.19 to 0.37)	2.348 (2.176 to 2.52)
Kiribati	0 (0 to 0)	0.74 (0.58 to 0.92)	1 (0 to 1)	1.01 (0.75 to 1.33)	1.098 (0.962 to 1.235)
Kuwait	4 (3 to 5)	0.77 (0.62 to 0.95)	22 (17 to 28)	1.04 (0.78 to 1.34)	0.99 (–0.552 to 2.557)
Kyrgyzstan	24 (20 to 28)	0.78 (0.65 to 0.93)	45 (37 to 54)	0.99 (0.82 to 1.18)	0.922 (0.286 to 1.562)
Lao People's Democratic Republic	12 (7 to 18)	0.59 (0.38 to 0.9)	26 (19 to 35)	0.67 (0.49 to 0.87)	0.465 (0.381 to 0.55)
Latvia	72 (61 to 83)	1.99 (1.69 to 2.29)	89 (70 to 112)	2.31 (1.83 to 2.92)	0.503 (–0.511 to 1.527)
Lebanon	19 (15 to 25)	0.89 (0.71 to 1.12)	112 (85 to 143)	2.17 (1.63 to 2.75)	3.11 (3.004 to 3.216)
Lesotho	4 (3 to 6)	0.45 (0.31 to 0.65)	10 (7 to 14)	0.87 (0.6 to 1.21)	2.314 (2.222 to 2.405)
Liberia	3 (2 to 4)	0.24 (0.15 to 0.35)	6 (3 to 10)	0.34 (0.19 to 0.52)	1.127 (0.927 to 1.328)

Libya	15 (9 to 22)	0.84 (0.49 to 1.25)	61 (43 to 85)	1.28 (0.91 to 1.77)	1.403 (1.061 to 1.746)
Lithuania	74 (61 to 85)	1.62 (1.35 to 1.87)	97 (75 to 122)	1.76 (1.35 to 2.23)	0.334 (–0.29 to 0.962)
Luxembourg	16 (13 to 19)	2.87 (2.39 to 3.35)	24 (19 to 29)	2.36 (1.9 to 2.88)	–0.68 (–0.968 to –0.391)
Madagascar	10 (6 to 15)	0.21 (0.13 to 0.31)	14 (8 to 23)	0.14 (0.08 to 0.22)	–1.346 (–1.604 to –1.087)
Malawi	7 (5 to 10)	0.22 (0.15 to 0.3)	20 (12 to 28)	0.3 (0.19 to 0.43)	1.111 (0.944 to 1.278)
Malaysia	30 (23 to 38)	0.36 (0.28 to 0.45)	145 (104 to 199)	0.59 (0.42 to 0.78)	1.68 (1.355 to 2.007)
Maldives	1 (0 to 1)	0.68 (0.45 to 1.02)	2 (2 to 3)	0.82 (0.63 to 1.03)	0.604 (0.374 to 0.835)
Mali	5 (3 to 8)	0.13 (0.08 to 0.19)	21 (12 to 33)	0.27 (0.16 to 0.4)	2.531 (2.348 to 2.715)
Malta	10 (8 to 12)	2.31 (1.96 to 2.71)	19 (15 to 23)	1.98 (1.6 to 2.41)	–0.518 (–0.902 to –0.132)
Marshall Islands	0 (0 to 0)	0.36 (0.26 to 0.47)	0 (0 to 0)	0.55 (0.39 to 0.76)	1.472 (1.198 to 1.746)
Mauritania	3 (2 to 5)	0.31 (0.2 to 0.46)	10 (6 to 14)	0.48 (0.32 to 0.68)	1.554 (1.417 to 1.691)
Mauritius	5 (4 to 6)	0.71 (0.57 to 0.84)	12 (9 to 17)	0.71 (0.52 to 0.94)	–0.086 (–1.04 to 0.878)
Mexico	484 (394 to 569)	1.23 (1.01 to 1.44)	832 (628 to 1063)	0.74 (0.56 to 0.94)	–1.695 (–1.996 to –1.392)
Micronesia (Federated States of)	0 (0 to 0)	0.8 (0.58 to 1.06)	1 (1 to 1)	1.26 (0.81 to 1.73)	1.56 (1.512 to 1.608)
Monaco	3 (2 to 3)	3.63 (2.69 to 4.75)	4 (3 to 6)	4.44 (3.31 to 5.76)	0.694 (0.609 to 0.78)
Mongolia	5 (4 to 7)	0.52 (0.38 to 0.66)	24 (17 to 32)	1.08 (0.8 to 1.42)	2.581 (2.212 to 2.951)
Montenegro	15 (12 to 18)	2.36 (1.91 to 2.89)	39 (32 to 48)	3.86 (3.11 to 4.71)	1.737 (1.538 to 1.937)
Morocco	40 (29 to 54)	0.3 (0.22 to 0.41)	139 (91 to 197)	0.46 (0.3 to 0.65)	1.427 (1.311 to 1.542)
Mozambique	10 (7 to 14)	0.2 (0.13 to 0.27)	32 (19 to 50)	0.33 (0.2 to 0.52)	1.86 (1.644 to 2.076)
Myanmar	161 (112 to 224)	0.76 (0.54 to 1.03)	344 (251 to 458)	0.83 (0.61 to 1.1)	0.327 (0.257 to 0.397)
Namibia	3 (2 to 3)	0.38 (0.28 to 0.51)	8 (6 to 11)	0.66 (0.48 to 0.9)	1.955 (1.706 to 2.204)
Nauru	0 (0 to 0)	1.04 (0.73 to 1.44)	0 (0 to 0)	1.41 (0.92 to 1.98)	1.052 (0.876 to 1.228)
Nepal	22 (11 to 35)	0.29 (0.14 to 0.48)	125 (69 to 190)	0.65 (0.36 to 0.98)	2.848 (2.762 to 2.933)
Netherlands	752 (660 to 842)	3.71 (3.26 to 4.13)	1165 (985 to 1372)	3.28 (2.78 to 3.83)	–0.399 (–0.563 to –0.234)
New Zealand	90 (78 to 103)	2.26 (1.95 to 2.58)	124 (103 to 149)	1.55 (1.29 to 1.86)	–1.29 (–1.543 to –1.036)
Nicaragua	5 (4 to 6)	0.33 (0.25 to 0.42)	22 (16 to 29)	0.54 (0.4 to 0.71)	1.604 (1.089 to 2.121)

Niger	1 (1 to 2)	0.05 (0.03 to 0.07)	6 (3 to 10)	0.09 (0.05 to 0.14)	2.221 (2.128 to 2.314)
Nigeria	48 (30 to 75)	0.12 (0.08 to 0.18)	120 (79 to 174)	0.16 (0.11 to 0.23)	0.978 (0.723 to 1.234)
Niue	0 (0 to 0)	0.69 (0.49 to 0.93)	0 (0 to 0)	1.03 (0.73 to 1.43)	1.411 (1.311 to 1.511)
North Macedonia	39 (33 to 46)	2.09 (1.77 to 2.47)	110 (84 to 143)	3.34 (2.55 to 4.32)	1.557 (1.423 to 1.692)
Northern Mariana Islands	0 (0 to 0)	0.73 (0.56 to 0.95)	1 (1 to 1)	1.34 (1.01 to 1.69)	2.086 (1.765 to 2.408)
Norway	165 (140 to 189)	2.38 (2.06 to 2.71)	161 (136 to 192)	1.68 (1.41 to 1.99)	–1.209 (–1.394 to –1.023)
Oman	2 (1 to 3)	0.29 (0.18 to 0.42)	7 (5 to 10)	0.53 (0.41 to 0.68)	2.084 (1.668 to 2.501)
Pakistan	193 (148 to 242)	0.36 (0.28 to 0.45)	460 (330 to 637)	0.48 (0.35 to 0.66)	0.976 (0.712 to 1.242)
Palau	0 (0 to 0)	1.11 (0.76 to 1.58)	0 (0 to 0)	1.36 (0.93 to 1.89)	0.704 (0.622 to 0.786)
Palestine	6 (3 to 9)	0.71 (0.42 to 1.08)	28 (22 to 35)	1.3 (1.02 to 1.61)	2.138 (1.738 to 2.539)
Panama	6 (5 to 7)	0.4 (0.33 to 0.48)	20 (14 to 27)	0.48 (0.34 to 0.65)	0.535 (0.018 to 1.054)
Papua New Guinea	4 (2 to 7)	0.24 (0.14 to 0.38)	13 (8 to 20)	0.29 (0.18 to 0.44)	0.706 (0.637 to 0.776)
Paraguay	14 (11 to 17)	0.66 (0.53 to 0.81)	73 (53 to 99)	1.4 (1.02 to 1.9)	2.64 (2.226 to 3.055)
Peru	15 (10 to 21)	0.13 (0.09 to 0.18)	88 (56 to 131)	0.28 (0.18 to 0.42)	2.721 (2.238 to 3.206)
Philippines	224 (180 to 271)	0.83 (0.68 to 0.99)	689 (525 to 882)	0.97 (0.76 to 1.23)	0.581 (0.333 to 0.829)
Poland	1290 (1131 to 1442)	2.92 (2.56 to 3.26)	2104 (1729 to 2554)	2.97 (2.44 to 3.59)	–0.035 (–0.221 to 0.15)
Portugal	201 (171 to 235)	1.41 (1.2 to 1.65)	293 (248 to 345)	1.29 (1.09 to 1.52)	–0.298 (–0.473 to –0.122)
Puerto Rico	12 (10 to 15)	0.33 (0.27 to 0.41)	64 (46 to 89)	0.87 (0.62 to 1.21)	3.381 (2.376 to 4.397)
Qatar	1 (0 to 1)	0.72 (0.51 to 1.03)	6 (4 to 9)	1.11 (0.75 to 1.58)	1.268 (–0.463 to 3.03)
Republic of Korea	581 (502 to 661)	2.02 (1.73 to 2.31)	1510 (1249 to 1784)	1.7 (1.4 to 2.01)	–0.698 (–1.04 to –0.355)
Republic of Moldova	58 (48 to 70)	1.27 (1.04 to 1.53)	101 (82 to 124)	1.73 (1.4 to 2.12)	0.974 (–0.776 to 2.755)
Romania	495 (419 to 568)	1.71 (1.45 to 1.95)	838 (653 to 1050)	2.32 (1.81 to 2.92)	1.131 (0.486 to 1.78)
Russian Federation	2755 (2334 to 3167)	1.48 (1.25 to 1.7)	4350 (3561 to 5275)	1.84 (1.5 to 2.22)	0.994 (–0.079 to 2.078)
Rwanda	11 (7 to 16)	0.44 (0.3 to 0.61)	33 (24 to 44)	0.67 (0.5 to 0.88)	1.429 (1.264 to 1.595)
Saint Kitts and Nevis	0 (0 to 0)	0.16 (0.13 to 0.21)	1 (0 to 1)	0.83 (0.65 to 1.09)	5.669 (4.946 to 6.396)
Saint Lucia	0 (0 to 0)	0.27 (0.22 to 0.33)	2 (2 to 3)	0.93 (0.72 to 1.2)	4.445 (3.689 to 5.206)

Saint Vincent and the Grenadines	0 (0 to 0)	0.18 (0.14 to 0.22)	1 (1 to 1)	0.65 (0.49 to 0.84)	4.662 (3.935 to 5.393)
Samoa	1 (1 to 1)	1.08 (0.86 to 1.35)	2 (1 to 2)	1.09 (0.83 to 1.47)	0.029 (–0.1 to 0.159)
San Marino	1 (1 to 1)	2.5 (1.99 to 3.11)	2 (1 to 2)	2.42 (1.48 to 3.71)	–0.081 (–0.189 to 0.027)
Sao Tome and Principe	0 (0 to 0)	0.07 (0.05 to 0.1)	0 (0 to 0)	0.19 (0.11 to 0.3)	3.516 (3.362 to 3.671)
Saudi Arabia	11 (7 to 16)	0.2 (0.12 to 0.3)	107 (77 to 142)	0.65 (0.48 to 0.84)	4.117 (3.931 to 4.303)
Senegal	8 (5 to 11)	0.24 (0.16 to 0.33)	23 (16 to 32)	0.32 (0.22 to 0.43)	0.89 (0.329 to 1.453)
Serbia	218 (181 to 261)	1.87 (1.55 to 2.21)	521 (400 to 668)	3.2 (2.45 to 4.11)	1.901 (1.615 to 2.189)
Seychelles	1 (0 to 1)	1.02 (0.78 to 1.28)	1 (1 to 2)	1.32 (1.04 to 1.63)	0.858 (0.365 to 1.355)
Sierra Leone	6 (4 to 8)	0.31 (0.2 to 0.43)	12 (8 to 18)	0.37 (0.23 to 0.53)	0.589 (0.419 to 0.76)
Singapore	23 (19 to 27)	1.09 (0.91 to 1.29)	59 (49 to 70)	0.76 (0.63 to 0.91)	–1.267 (–1.53 to –1.004)
Slovakia	132 (113 to 153)	2.18 (1.87 to 2.52)	206 (154 to 272)	2.2 (1.65 to 2.89)	–0.028 (–0.317 to 0.261)
Slovenia	55 (41 to 72)	2.21 (1.66 to 2.92)	105 (78 to 144)	2.44 (1.82 to 3.32)	0.227 (–0.245 to 0.701)
Solomon Islands	1 (0 to 1)	0.46 (0.31 to 0.65)	2 (1 to 3)	0.67 (0.46 to 0.92)	1.251 (1.131 to 1.372)
Somalia	4 (2 to 8)	0.19 (0.09 to 0.34)	9 (4 to 16)	0.15 (0.06 to 0.26)	–0.869 (–0.951 to –0.786)
South Africa	242 (186 to 322)	1.22 (0.93 to 1.63)	414 (332 to 505)	0.97 (0.78 to 1.18)	–0.604 (–0.822 to –0.384)
South Sudan	6 (4 to 10)	0.27 (0.17 to 0.46)	9 (5 to 14)	0.26 (0.15 to 0.41)	–0.125 (–0.22 to –0.029)
Spain	1020 (884 to 1156)	1.86 (1.62 to 2.1)	1921 (1648 to 2239)	2.14 (1.84 to 2.48)	0.472 (0.345 to 0.599)
Sri Lanka	30 (21 to 39)	0.31 (0.22 to 0.4)	76 (48 to 112)	0.3 (0.2 to 0.44)	–0.035 (–0.38 to 0.311)
Sudan	20 (11 to 36)	0.22 (0.13 to 0.4)	104 (62 to 180)	0.61 (0.36 to 1.06)	3.537 (3.4 to 3.674)
Suriname	1 (1 to 1)	0.32 (0.25 to 0.38)	8 (6 to 10)	1.32 (1.01 to 1.7)	5.213 (4.449 to 5.982)
Sweden	454 (382 to 524)	2.91 (2.47 to 3.32)	513 (428 to 603)	2.31 (1.95 to 2.69)	–0.792 (–1.002 to –0.582)
Switzerland	182 (155 to 211)	1.73 (1.48 to 1.99)	410 (348 to 492)	2.29 (1.95 to 2.7)	0.951 (0.607 to 1.296)
Syrian Arab Republic	21 (15 to 29)	0.43 (0.3 to 0.57)	86 (62 to 119)	0.74 (0.54 to 1)	1.884 (1.491 to 2.279)
Taiwan (Province of China)	158 (134 to 180)	0.97 (0.83 to 1.11)	566 (426 to 769)	1.4 (1.05 to 1.9)	1.322 (1.06 to 1.585)
Tajikistan	17 (11 to 27)	0.65 (0.4 to 1.02)	28 (21 to 36)	0.67 (0.49 to 0.9)	0.074 (–0.5 to 0.651)
Thailand	270 (216 to 333)	0.81 (0.67 to 0.99)	675 (478 to 917)	0.67 (0.47 to 0.91)	–0.738 (–1.101 to –0.374)

Timor-Leste	1 (0 to 1)	0.31 (0.17 to 0.47)	4 (2 to 5)	0.48 (0.3 to 0.68)	1.582 (1.326 to 1.838)
Togo	4 (3 to 5)	0.32 (0.23 to 0.43)	15 (10 to 22)	0.45 (0.32 to 0.65)	1.296 (1.044 to 1.549)
Tokelau	0 (0 to 0)	0.51 (0.35 to 0.7)	0 (0 to 0)	0.78 (0.51 to 1.08)	1.462 (1.403 to 1.522)
Tonga	0 (0 to 1)	0.77 (0.54 to 1.03)	1 (1 to 1)	1.02 (0.7 to 1.41)	0.945 (0.453 to 1.439)
Trinidad and Tobago	2 (2 to 3)	0.27 (0.22 to 0.32)	16 (11 to 22)	0.85 (0.6 to 1.18)	4.214 (3.245 to 5.193)
Tunisia	24 (17 to 31)	0.5 (0.37 to 0.66)	102 (71 to 141)	0.84 (0.59 to 1.15)	1.793 (1.612 to 1.974)
Turkey	519 (397 to 679)	1.46 (1.12 to 1.9)	1731 (1348 to 2196)	1.96 (1.53 to 2.5)	0.968 (0.622 to 1.315)
Turkmenistan	5 (4 to 6)	0.25 (0.21 to 0.3)	21 (16 to 28)	0.54 (0.41 to 0.7)	2.847 (1.623 to 4.085)
Tuvalu	0 (0 to 0)	0.56 (0.41 to 0.73)	0 (0 to 0)	0.79 (0.55 to 1.1)	1.203 (1.127 to 1.28)
Uganda	12 (7 to 17)	0.2 (0.12 to 0.28)	48 (31 to 70)	0.39 (0.25 to 0.55)	2.354 (2.206 to 2.501)
Ukraine	780 (626 to 971)	1.07 (0.86 to 1.32)	1335 (1034 to 1657)	1.8 (1.4 to 2.25)	1.853 (–0.139 to 3.884)
United Arab Emirates	5 (2 to 9)	1.28 (0.63 to 2.34)	100 (46 to 165)	2.61 (1.32 to 3.99)	2.449 (1.99 to 2.91)
United Kingdom	3025 (2663 to 3387)	3.24 (2.85 to 3.62)	3487 (2966 to 4103)	2.67 (2.3 to 3.12)	–0.667 (–0.791 to –0.542)
United Republic of Tanzania	44 (30 to 59)	0.44 (0.3 to 0.58)	124 (81 to 177)	0.56 (0.37 to 0.79)	0.869 (0.662 to 1.076)
United States of America	9990 (8419 to 11595)	3.07 (2.61 to 3.54)	15793 (13072 to 18776)	2.76 (2.31 to 3.26)	–0.363 (–0.596 to –0.129)
United States Virgin Islands	0 (0 to 1)	0.5 (0.38 to 0.65)	3 (2 to 4)	1.37 (1.02 to 1.83)	3.558 (3.337 to 3.78)
Uruguay	102 (87 to 119)	2.57 (2.2 to 2.98)	178 (150 to 208)	3.34 (2.81 to 3.88)	0.834 (0.515 to 1.154)
Uzbekistan	16 (12 to 23)	0.15 (0.11 to 0.21)	95 (72 to 122)	0.5 (0.39 to 0.63)	4.504 (4.059 to 4.952)
Vanuatu	0 (0 to 0)	0.32 (0.22 to 0.46)	1 (0 to 1)	0.4 (0.27 to 0.55)	0.691 (0.517 to 0.865)
Venezuela (Bolivarian Republic of)	34 (27 to 39)	0.37 (0.3 to 0.43)	239 (169 to 328)	0.84 (0.6 to 1.16)	2.727 (1.265 to 4.21)
Viet Nam	115 (84 to 149)	0.29 (0.22 to 0.38)	606 (439 to 822)	0.69 (0.51 to 0.93)	2.97 (2.866 to 3.074)
Yemen	13 (7 to 22)	0.28 (0.15 to 0.46)	59 (43 to 82)	0.48 (0.35 to 0.66)	1.89 (1.751 to 2.028)
Zambia	10 (6 to 14)	0.41 (0.27 to 0.57)	31 (19 to 48)	0.57 (0.36 to 0.84)	1.158 (1.067 to 1.249)
Zimbabwe	35 (28 to 44)	0.99 (0.8 to 1.2)	66 (47 to 86)	1.09 (0.8 to 1.43)	0.403 (0.131 to 0.677)

ASDR, age-standardized death rate; AAPC, average annual percentage change.

Table S3 The DALY and its change trends in smoking-related pancreatic cancer from 1990 to 2019 among all countries/territories

Country/region	DALYs in 1990	Age-standardized DALY rate in 1990	DALYs in 2019	Age-standardized DALY rate in 2019	AAPC and 95% CI
Afghanistan	208 (97 to 422)	2.8 (1.35 to 5.56)	819 (460 to 1410)	6.03 (3.66 to 9.96)	2.691 (2.437 to 2.944)
Albania	448 (379 to 529)	21.07 (18.01 to 24.87)	1648 (1174 to 2226)	38.23 (27.18 to 51.62)	1.957 (1.521 to 2.395)
Algeria	1003 (750 to 1311)	8.32 (6.36 to 10.77)	4762 (3539 to 6134)	14.3 (10.7 to 18.42)	1.879 (1.738 to 2.02)
American Samoa	4 (3 to 5)	17.03 (12.69 to 21.68)	12 (9 to 16)	24.54 (18.21 to 31.97)	1.343 (0.658 to 2.033)
Andorra	34 (25 to 49)	59.65 (43.4 to 85.04)	75 (54 to 100)	53.49 (38.75 to 71.18)	–0.349 (–0.486 to –0.213)
Angola	265 (156 to 422)	6.34 (3.88 to 9.75)	973 (644 to 1400)	8.16 (5.65 to 11.37)	0.855 (0.647 to 1.064)
Antigua and Barbuda	2 (1 to 2)	3.27 (2.6 to 4.05)	16 (12 to 21)	15.52 (11.44 to 20.05)	5.537 (4.618 to 6.464)
Argentina	21018 (17866 to 24131)	63.68 (54.11 to 73.08)	34922 (29715 to 40954)	65.57 (55.81 to 76.65)	0.163 (–0.17 to 0.497)
Armenia	1110 (884 to 1378)	38.17 (30.62 to 47.19)	2181 (1690 to 2705)	51.68 (40.21 to 64.11)	1.097 (0.701 to 1.494)
Australia	9370 (8260 to 10466)	47.86 (42.3 to 53.37)	12715 (10758 to 14952)	32.55 (27.47 to 38.34)	–1.328 (–1.495 to –1.161)
Austria	6592 (5711 to 7515)	58.43 (50.54 to 66.22)	9918 (8597 to 11311)	60.36 (52.49 to 68.87)	0.087 (–0.088 to 0.263)
Azerbaijan	872 (664 to 1139)	16.27 (12.59 to 21.04)	3267 (2454 to 4325)	31.65 (24.17 to 40.65)	2.312 (2.051 to 2.575)
Bahamas	8 (6 to 10)	4.98 (3.86 to 6.26)	43 (32 to 56)	10.56 (7.83 to 13.81)	2.633 (2.338 to 2.93)
Bahrain	52 (40 to 67)	30.51 (23.7 to 38.4)	233 (166 to 317)	24.54 (18.27 to 32.99)	–0.824 (–1.487 to –0.156)
Bangladesh	3254 (1779 to 5011)	7.07 (3.9 to 10.77)	9819 (5373 to 15393)	7.55 (4.16 to 11.84)	0.345 (0.027 to 0.665)
Barbados	9 (7 to 11)	3.42 (2.73 to 4.19)	72 (53 to 95)	14.7 (10.95 to 19.43)	5.121 (4.652 to 5.591)
Belarus	3918 (3323 to 4567)	29.64 (25.14 to	6263 (4648 to 8557)	40.34 (29.96 to 55.47)	1.139 (0.634 to 1.647)

		34.61)			
Belgium	8855 (7745 to 9992)	58.59 (51.28 to 65.94)	11058 (9567 to 12779)	52.6 (45.61 to 60.37)	–0.364 (–0.637 to –0.091)
Belize	5 (4 to 6)	5.21 (4.15 to 6.47)	46 (35 to 60)	16.5 (12.58 to 21.17)	4.17 (3.649 to 4.694)
Benin	104 (70 to 145)	5.28 (3.57 to 7.31)	337 (211 to 504)	7.07 (4.47 to 10.32)	1.008 (0.745 to 1.272)
Bermuda	6 (4 to 7)	9.24 (7.08 to 11.68)	37 (27 to 49)	28.62 (21.22 to 38.17)	3.956 (3.439 to 4.475)
Bhutan	9 (4 to 15)	3.47 (1.56 to 5.83)	32 (17 to 50)	5.84 (3.16 to 9.01)	1.82 (1.724 to 1.915)
Bolivia (Plurinational State of)	262 (166 to 371)	8.11 (5.18 to 11.39)	858 (507 to 1309)	9.59 (5.68 to 14.58)	0.575 (0.37 to 0.781)
Bosnia and Herzegovina	2057 (1759 to 2384)	47.53 (40.64 to 54.85)	4332 (3277 to 5669)	72.23 (54.46 to 94.3)	1.431 (1.268 to 1.595)
Botswana	81 (55 to 112)	13.96 (9.78 to 19.18)	337 (220 to 492)	24.61 (16.54 to 35.02)	1.982 (1.77 to 2.194)
Brazil	35113 (30702 to 39391)	38.73 (33.97 to 43.64)	69400 (58076 to 80534)	28.89 (24.21 to 33.63)	–1.01 (–1.17 to –0.85)
Brunei Darussalam	37 (29 to 46)	38.1 (30.08 to 47.69)	97 (76 to 120)	32.53 (25.47 to 40.17)	–0.494 (–0.907 to –0.079)
Bulgaria	7452 (6538 to 8419)	58.21 (51.27 to 65.89)	11088 (8359 to 14432)	86.28 (64.62 to 112.89)	1.491 (0.88 to 2.106)
Burkina Faso	126 (65 to 208)	2.72 (1.42 to 4.46)	353 (191 to 562)	3.67 (2.01 to 5.79)	1.073 (0.914 to 1.232)
Burundi	169 (104 to 254)	7.12 (4.45 to 10.62)	203 (117 to 321)	4.27 (2.51 to 6.63)	–1.758 (–2.015 to –1.5)
Cabo Verde	6 (4 to 8)	2.62 (1.92 to 3.39)	59 (40 to 82)	14.24 (9.74 to 19.61)	6.047 (5.441 to 6.656)
Cambodia	584 (378 to 864)	12.88 (8.43 to 18.68)	1996 (1494 to 2523)	16.76 (12.66 to 20.78)	0.92 (0.837 to 1.003)
Cameroon	375 (228 to 573)	7.91 (4.96 to 11.95)	1430 (833 to 2296)	11.54 (7 to 17.97)	1.329 (1.227 to 1.432)
Canada	21958 (19453 to 24458)	67.98 (60.32 to 75.78)	29613 (24947 to 34835)	44 (37.3 to 51.57)	–1.471 (–1.617 to –1.326)
Central African Republic	62 (31 to 109)	4.83 (2.56 to 8.21)	94 (49 to 161)	3.89 (2.17 to 6.34)	–0.778 (–0.965 to –0.59)
Chad	81 (49 to 118)	2.87 (1.77 to 4.15)	261 (160 to 389)	4.68 (2.92 to 6.88)	1.727 (1.552 to 1.903)
Chile	4075 (3402 to 4747)	39.58 (32.98 to	8985 (7351 to 10659)	37.07 (30.3 to 43.94)	–0.179 (–0.647 to 0.292)

		46.21)			
China	142185 (109126 to 180266)	15.99 (12.5 to 20)	612359 (474505 to 779859)	29.29 (22.86 to 37.08)	2.076 (1.855 to 2.297)
Colombia	4502 (3580 to 5365)	25.33 (20.25 to 30.07)	6520 (4571 to 9135)	12.41 (8.7 to 17.4)	–2.56 (–2.799 to –2.321)
Comoros	16 (8 to 25)	7.38 (3.75 to 10.9)	38 (22 to 56)	7.78 (4.64 to 11.46)	0.319 (0.036 to 0.602)
Congo	84 (46 to 135)	7.44 (4.31 to 11.67)	259 (145 to 423)	9.42 (5.57 to 14.96)	0.85 (0.676 to 1.024)
Cook Islands	2 (2 to 3)	18.68 (13.28 to 24.91)	5 (4 to 7)	20.95 (15.2 to 28.01)	0.351 (0.116 to 0.585)
Costa Rica	254 (211 to 304)	14.68 (12.15 to 17.69)	1045 (742 to 1433)	20.22 (14.4 to 27.78)	1.288 (0.268 to 2.318)
Croatia	4556 (4007 to 5140)	69.29 (60.74 to 78.03)	4866 (3662 to 6325)	58.75 (43.97 to 76.81)	–0.455 (–1.121 to 0.214)
Cuba	1134 (975 to 1304)	11.08 (9.52 to 12.73)	6603 (5122 to 8183)	35.22 (27.36 to 43.8)	4.269 (3.663 to 4.879)
Cyprus	201 (168 to 240)	24.03 (20.22 to 28.49)	894 (734 to 1068)	45.76 (37.76 to 54.55)	2.325 (2.094 to 2.556)
Czechia	11440 (9955 to 13051)	83.67 (72.9 to 95.29)	14318 (10987 to 18225)	70.21 (53.59 to 89.06)	–0.642 (–1.277 to –0.002)
Côte d'Ivoire	276 (181 to 399)	6.31 (4.25 to 8.85)	1326 (870 to 1862)	11.92 (8.08 to 16.38)	2.227 (2.018 to 2.435)
Democratic People's Republic of Korea	2075 (1384 to 3056)	11.5 (7.98 to 16.48)	6047 (4193 to 8190)	18.14 (12.66 to 24.31)	1.59 (1.512 to 1.668)
Democratic Republic of the Congo	754 (415 to 1159)	4.48 (2.5 to 6.8)	1324 (735 to 2040)	3.5 (1.97 to 5.31)	–0.849 (–1.032 to –0.665)
Denmark	5138 (4546 to 5708)	65.47 (57.98 to 72.59)	6984 (5953 to 8125)	61.98 (52.79 to 71.93)	–0.183 (–0.445 to 0.08)
Djibouti	11 (7 to 18)	7.81 (4.7 to 12.2)	89 (48 to 142)	14.31 (8.24 to 21.85)	2.094 (1.852 to 2.337)
Dominica	4 (3 to 5)	5.35 (4.18 to 6.73)	17 (12 to 24)	18.93 (13.53 to 26.14)	4.45 (4.092 to 4.809)

Dominican Republic	236 (189 to 291)	6.44 (5.15 to 7.96)	1488 (952 to 2158)	16.14 (10.35 to 23.36)	3.25 (2.882 to 3.619)
Ecuador	324 (249 to 398)	6.17 (4.79 to 7.52)	1891 (1328 to 2562)	12.52 (8.84 to 16.9)	2.335 (1.913 to 2.759)
Egypt	2743 (2188 to 3370)	8.68 (6.99 to 10.53)	14882 (9629 to 22691)	21.24 (14.05 to 31.86)	3.224 (2.796 to 3.653)
El Salvador	133 (102 to 164)	4.51 (3.48 to 5.54)	574 (385 to 823)	9.95 (6.66 to 14.29)	2.914 (2.238 to 3.594)
Equatorial Guinea	9 (4 to 19)	4.39 (1.92 to 8.62)	48 (23 to 88)	9.8 (4.91 to 17.43)	2.834 (2.593 to 3.076)
Eritrea	38 (21 to 58)	3.13 (1.8 to 4.76)	108 (54 to 187)	3.41 (1.78 to 5.63)	0.27 (0.119 to 0.42)
Estonia	1103 (938 to 1262)	53.58 (45.31 to 61.24)	1419 (1061 to 1848)	59.14 (44.02 to 78.27)	0.396 (–0.609 to 1.411)
Eswatini	32 (19 to 50)	11.36 (6.85 to 17.41)	70 (41 to 115)	12.8 (7.74 to 20.26)	0.394 (0.054 to 0.735)
Ethiopia	339 (127 to 692)	1.66 (0.64 to 3.35)	485 (244 to 854)	1.22 (0.61 to 2.14)	–1.06 (–1.206 to –0.914)
Fiji	58 (42 to 75)	15.51 (11.56 to 19.9)	125 (84 to 178)	15.91 (10.87 to 22.45)	0.072 (0.009 to 0.135)
Finland	4221 (3612 to 4882)	60.32 (51.88 to 69.16)	5481 (4584 to 6564)	47.92 (40.01 to 57.14)	–0.779 (–0.985 to –0.573)
France	35645 (30526 to 40436)	45.78 (39.19 to 51.81)	59797 (50238 to 69987)	49.24 (41.35 to 57.75)	0.195 (–0.07 to 0.461)
Gabon	42 (24 to 71)	7.06 (4.14 to 11.92)	152 (82 to 258)	13.65 (7.63 to 22.74)	2.324 (2.124 to 2.525)
Gambia	17 (11 to 25)	4.76 (3.08 to 6.83)	65 (39 to 103)	6.81 (4.19 to 10.57)	1.236 (0.742 to 1.732)
Georgia	1247 (980 to 1618)	19.08 (15.03 to 24.57)	1863 (1456 to 2332)	33.34 (25.93 to 41.72)	1.997 (1.016 to 2.987)
Germany	67715 (58978 to 77506)	55.1 (48.06 to 62.73)	105274 (90860 to 121941)	60.72 (52.65 to 70.49)	0.36 (0.132 to 0.589)
Ghana	393 (258 to 546)	6.31 (4.21 to 8.7)	1376 (831 to 2141)	8.7 (5.29 to 13.41)	1.155 (0.867 to 1.443)
Greece	9341 (8231 to 10491)	61.12 (53.87 to 68.52)	15003 (13111 to 17193)	72.99 (64.01 to 83.4)	0.612 (0.384 to 0.84)
Greenland	52 (43 to 62)	142.76 (118.69 to 169.94)	109 (84 to 138)	146.47 (114.11 to 184.15)	0.145 (–0.248 to 0.54)
Grenada	3 (2 to 4)	4.57 (3.57 to 5.75)	29 (21 to 37)	24.58 (18.38 to 31.24)	6.052 (5.348 to 6.76)

Guam	14 (10 to 19)	17.42 (12.74 to 22.38)	43 (31 to 56)	21.77 (16.05 to 28.68)	0.79 (0.399 to 1.183)
Guatemala	158 (106 to 210)	4.24 (2.89 to 5.59)	938 (613 to 1379)	8.56 (5.62 to 12.56)	2.543 (1.887 to 3.202)
Guinea	65 (42 to 93)	1.91 (1.23 to 2.72)	191 (124 to 265)	3.45 (2.28 to 4.75)	2.091 (1.974 to 2.207)
Guinea-Bissau	18 (9 to 31)	4.22 (2.19 to 7.12)	34 (18 to 54)	4.49 (2.53 to 7.09)	0.219 (0.166 to 0.272)
Guyana	20 (15 to 26)	5.3 (4.04 to 6.69)	103 (69 to 146)	15.27 (10.39 to 21.36)	3.769 (3.372 to 4.167)
Haiti	96 (60 to 139)	2.76 (1.77 to 3.98)	291 (176 to 445)	4.02 (2.44 to 6.09)	1.311 (1.177 to 1.444)
Honduras	145 (102 to 192)	7.05 (4.95 to 9.21)	664 (359 to 1053)	11.01 (5.96 to 17.42)	1.609 (0.818 to 2.407)
Hungary	10729 (9414 to 12087)	73.27 (64.14 to 82.96)	14910 (11712 to 18750)	82.52 (64.4 to 104.43)	0.52 (0.072 to 0.971)
Iceland	173 (147 to 200)	62.24 (53.02 to 71.74)	216 (178 to 260)	40.43 (33.63 to 48.57)	–1.503 (–1.804 to –1.201)
India	23455 (15662 to 32725)	5.2 (3.53 to 7.13)	83185 (60151 to 106890)	7.3 (5.32 to 9.26)	1.281 (0.775 to 1.79)
Indonesia	8483 (6082 to 10902)	8.24 (6.14 to 10.44)	42937 (27513 to 65342)	19.2 (12.34 to 29.18)	2.976 (2.878 to 3.073)
Iran (Islamic Republic of)	2514 (1848 to 3299)	8.85 (6.55 to 11.64)	12136 (10040 to 14331)	16.3 (13.48 to 19.29)	2.098 (1.807 to 2.39)
Iraq	1424 (925 to 2016)	18.16 (11.8 to 25.59)	6992 (5010 to 9277)	29.69 (21.61 to 38.54)	1.657 (1.474 to 1.841)
Ireland	2926 (2582 to 3258)	71.25 (62.82 to 79.12)	3499 (2936 to 4096)	47.14 (39.44 to 55.12)	–1.409 (–1.627 to –1.19)
Israel	2899 (2532 to 3306)	60.05 (52.47 to 68.07)	5531 (4702 to 6510)	49.14 (41.89 to 57.87)	–0.715 (–1.011 to –0.419)
Italy	48815 (42716 to 55066)	55.86 (48.75 to 62.58)	62981 (54519 to 72060)	49.07 (42.26 to 55.82)	–0.517 (–0.623 to –0.41)
Jamaica	90 (74 to 108)	5.24 (4.27 to 6.24)	521 (379 to 713)	17.66 (12.92 to 24.17)	4.341 (2.507 to 6.208)
Japan	102312 (90050 to 114724)	58.87 (51.92 to 66.05)	131283 (112819 to 150162)	42.95 (36.98 to 48.68)	–1.117 (–1.227 to –1.008)
Jordan	222 (175 to 279)	16.13 (12.74 to	1922 (1454 to 2467)	28.22 (21.57 to 35.76)	1.932 (1.557 to 2.309)

		20.32)			
Kazakhstan	635 (510 to 765)	4.82 (3.88 to 5.8)	5538 (4330 to 6806)	29.39 (23.14 to 35.84)	6.579 (5.933 to 7.228)
Kenya	241 (130 to 385)	2.95 (1.62 to 4.66)	1278 (852 to 1823)	5.81 (3.97 to 8.21)	2.352 (2.158 to 2.547)
Kiribati	6 (5 to 8)	16.77 (13.06 to 21.09)	15 (11 to 20)	21.63 (15.94 to 29.18)	0.884 (0.744 to 1.025)
Kuwait	109 (86 to 133)	16.86 (13.66 to 20.64)	508 (383 to 660)	19.78 (14.92 to 25.89)	0.513 (–1.046 to 2.097)
Kyrgyzstan	647 (529 to 775)	20.76 (17.04 to 24.79)	1192 (967 to 1465)	24.25 (19.81 to 29.53)	0.642 (–0.018 to 1.307)
Lao People's Democratic Republic	302 (185 to 491)	13.83 (8.71 to 22.4)	636 (450 to 861)	14.4 (10.42 to 19.29)	0.145 (0.089 to 0.201)
Latvia	1808 (1534 to 2099)	50.48 (42.71 to 58.56)	2005 (1576 to 2543)	56.76 (44.17 to 71.99)	0.545 (–0.531 to 1.632)
Lebanon	473 (361 to 608)	19.92 (15.5 to 25.45)	2413 (1788 to 3118)	46.79 (34.48 to 60.58)	2.993 (2.889 to 3.097)
Lesotho	102 (66 to 148)	10.13 (6.71 to 14.57)	255 (162 to 377)	19.61 (12.88 to 28.08)	2.306 (2.208 to 2.404)
Liberia	64 (39 to 95)	5.53 (3.38 to 8.25)	153 (82 to 248)	7.56 (4.17 to 11.93)	1.037 (0.861 to 1.213)
Libya	370 (216 to 556)	19.29 (11.19 to 28.85)	1551 (1076 to 2177)	29.1 (20.46 to 40.59)	1.399 (1.026 to 1.772)
Lithuania	1889 (1544 to 2196)	41.64 (34.08 to 48.47)	2215 (1669 to 2809)	43.42 (32.44 to 55.38)	0.172 (–0.519 to 0.868)
Luxembourg	343 (287 to 398)	63.73 (53.53 to 73.72)	457 (373 to 551)	47.02 (38.23 to 56.85)	–1.054 (–1.416 to –0.689)
Madagascar	264 (157 to 387)	4.96 (2.98 to 7.21)	395 (214 to 635)	3.37 (1.9 to 5.32)	–1.355 (–1.575 to –1.134)
Malawi	170 (112 to 235)	4.52 (3.02 to 6.17)	453 (273 to 672)	6.43 (3.94 to 9.31)	1.204 (1.016 to 1.393)
Malaysia	730 (557 to 916)	7.89 (6.11 to 9.9)	3374 (2370 to 4678)	12.35 (8.8 to 16.9)	1.671 (1.367 to 1.977)
Maldives	13 (8 to 21)	14.28 (8.95 to 22.14)	48 (37 to 61)	16.01 (12.23 to 20.13)	0.337 (0.099 to 0.576)

Mali	132 (77 to 202)	3.03 (1.79 to 4.59)	522 (298 to 814)	5.97 (3.44 to 9.25)	2.374 (2.146 to 2.603)
Malta	223 (190 to 258)	51.44 (43.86 to 59.63)	384 (312 to 466)	44.1 (36.05 to 53.24)	–0.571 (–0.746 to –0.395)
Marshall Islands	2 (1 to 2)	8.91 (6.42 to 11.67)	5 (3 to 7)	13.37 (9.02 to 18.76)	1.392 (1.121 to 1.663)
Mauritania	81 (50 to 123)	7.8 (4.87 to 11.81)	245 (156 to 365)	11.35 (7.24 to 16.66)	1.278 (1.123 to 1.434)
Mauritius	128 (98 to 156)	16.71 (13.08 to 20.12)	293 (206 to 404)	15.99 (11.36 to 21.8)	–0.222 (–1.302 to 0.87)
Mexico	11011 (8665 to 13161)	25.94 (20.74 to 30.8)	17738 (12956 to 23225)	15.18 (11.2 to 19.81)	–1.782 (–2.066 to –1.497)
Micronesia (Federated States of)	9 (6 to 13)	19.36 (13.61 to 26.3)	23 (14 to 34)	30.04 (18.81 to 42.75)	1.517 (1.474 to 1.56)
Monaco	51 (38 to 66)	79.39 (59.58 to 103.55)	82 (61 to 107)	94.66 (70.57 to 122.93)	0.613 (0.53 to 0.695)
Mongolia	131 (93 to 172)	12.22 (8.8 to 15.94)	655 (442 to 920)	25.27 (18.12 to 34.14)	2.523 (2.165 to 2.882)
Montenegro	365 (297 to 449)	56.7 (46.13 to 69.63)	894 (717 to 1095)	89.28 (71.97 to 109.86)	1.683 (1.448 to 1.919)
Morocco	1034 (737 to 1406)	7.22 (5.18 to 9.76)	3505 (2296 to 4995)	10.55 (6.87 to 15.02)	1.313 (1.223 to 1.403)
Mozambique	241 (150 to 339)	4.11 (2.64 to 5.71)	766 (442 to 1227)	7.17 (4.18 to 11.16)	1.957 (1.764 to 2.149)
Myanmar	3988 (2708 to 5733)	16.87 (11.68 to 23.66)	7544 (5392 to 10178)	16.59 (12 to 22.46)	–0.059 (–0.131 to 0.012)
Namibia	53 (36 to 72)	7.44 (5.2 to 10.1)	157 (109 to 220)	12.04 (8.41 to 16.61)	1.716 (1.449 to 1.985)
Nauru	1 (1 to 1)	25.26 (17 to 35.37)	2 (1 to 2)	34.05 (21.19 to 48.79)	1.032 (0.86 to 1.205)
Nepal	511 (255 to 823)	5.74 (2.79 to 9.17)	2546 (1388 to 3881)	11.88 (6.55 to 18)	2.584 (2.488 to 2.681)
Netherlands	15474 (13852 to 17020)	79.1 (70.62 to 87.13)	21584 (18632 to 25088)	64.64 (55.84 to 74.74)	–0.661 (–0.811 to –0.511)
New Zealand	1883 (1638 to 2135)	47.92 (41.46 to 54.36)	2432 (2043 to 2883)	32.17 (27.12 to 37.83)	–1.362 (–1.617 to –1.107)
Nicaragua	112 (83 to 142)	7.32 (5.47 to 9.29)	488 (347 to 650)	11.08 (7.92 to 14.79)	1.32 (0.774 to 1.869)

Niger	33 (17 to 55)	1.11 (0.6 to 1.79)	153 (76 to 264)	1.89 (0.98 to 3.16)	1.943 (1.803 to 2.084)
Nigeria	1145 (667 to 1837)	2.59 (1.55 to 4.07)	2851 (1813 to 4196)	3.38 (2.18 to 4.91)	0.936 (0.767 to 1.106)
Niue	0 (0 to 0)	16.3 (11.37 to 22.28)	1 (0 to 1)	23.45 (16.5 to 32.55)	1.245 (1.122 to 1.367)
North Macedonia	1006 (847 to 1173)	50.85 (42.76 to 59.45)	2597 (1950 to 3408)	77.5 (58.4 to 102.07)	1.442 (1.21 to 1.675)
Northern Mariana Islands	3 (2 to 4)	16.94 (12.47 to 22.66)	18 (13 to 23)	30.32 (22.56 to 39.15)	2.048 (1.593 to 2.505)
Norway	3381 (2936 to 3841)	53.26 (46.51 to 60.08)	3329 (2810 to 3900)	36.71 (31.11 to 42.84)	–1.298 (–1.501 to –1.096)
Oman	47 (28 to 69)	6.68 (4.11 to 9.82)	187 (130 to 267)	10.74 (8.19 to 14.28)	1.581 (1.262 to 1.902)
Pakistan	4285 (3271 to 5406)	7.61 (5.82 to 9.59)	10834 (7547 to 15272)	10.06 (7.18 to 14)	0.962 (0.7 to 1.225)
Palau	3 (2 to 4)	25.96 (17.33 to 37.33)	7 (5 to 10)	30.75 (20.76 to 42.7)	0.591 (0.503 to 0.68)
Palestine	134 (79 to 211)	15.46 (9.19 to 24.07)	700 (542 to 883)	28.36 (22.37 to 35.47)	2.035 (1.548 to 2.525)
Panama	126 (101 to 150)	8.51 (6.89 to 10.18)	385 (273 to 534)	9.34 (6.63 to 13.01)	0.287 (–0.234 to 0.811)
Papua New Guinea	113 (61 to 184)	5.7 (3.19 to 9.17)	345 (205 to 540)	6.88 (4.19 to 10.58)	0.677 (0.579 to 0.775)
Paraguay	294 (235 to 358)	13.48 (10.84 to 16.37)	1492 (1044 to 2057)	27.29 (19.29 to 37.46)	2.467 (2.063 to 2.872)
Peru	356 (232 to 502)	2.98 (1.96 to 4.19)	1894 (1187 to 2854)	5.99 (3.75 to 9.01)	2.515 (2.017 to 3.016)
Philippines	5720 (4475 to 7000)	18.47 (14.75 to 22.31)	16699 (12408 to 21880)	20.95 (15.89 to 27.04)	0.485 (0.154 to 0.817)
Poland	31769 (27656 to 35324)	71.93 (62.77 to 79.96)	46738 (38285 to 57035)	68.68 (55.82 to 83.63)	–0.223 (–0.467 to 0.022)
Portugal	4613 (3917 to 5402)	33.16 (28 to 38.64)	6359 (5322 to 7487)	31.1 (25.91 to 36.53)	–0.216 (–0.48 to 0.05)
Puerto Rico	263 (209 to 324)	7.22 (5.73 to 8.88)	1241 (883 to 1713)	18.37 (12.87 to 25.47)	3.305 (2.211 to 4.411)
Qatar	18 (12 to 27)	15.79 (10.97 to	180 (110 to 267)	19.78 (13.32 to 27.74)	0.485 (–0.93 to 1.921)

		23.16)			
Republic of Korea	15532 (13304 to 17589)	47.93 (41.47 to 54.23)	30958 (25804 to 36422)	34.08 (28.37 to 40.02)	–1.224 (–1.584 to –0.862)
Republic of Moldova	1616 (1314 to 1922)	34.58 (28.31 to 41)	2718 (2173 to 3327)	47.53 (38.29 to 58.38)	1.019 (–0.949 to 3.025)
Romania	13106 (11056 to 15139)	44.81 (37.81 to 51.62)	20022 (15449 to 25289)	58.97 (45.29 to 74.51)	1.082 (0.464 to 1.704)
Russian Federation	76014 (64015 to 87991)	40.66 (34.38 to 46.94)	109870 (89464 to 133525)	47.45 (38.5 to 57.83)	0.762 (–0.44 to 1.978)
Rwanda	266 (166 to 386)	9.34 (6.01 to 13.24)	727 (514 to 995)	13 (9.5 to 17.33)	1.112 (0.92 to 1.305)
Saint Kitts and Nevis	1 (1 to 2)	3.55 (2.81 to 4.42)	13 (10 to 17)	18.18 (13.8 to 23.95)	5.707 (4.786 to 6.637)
Saint Lucia	5 (4 to 6)	6.14 (4.94 to 7.39)	45 (34 to 59)	20.62 (15.64 to 26.77)	4.39 (3.771 to 5.013)
Saint Vincent and the Grenadines	3 (2 to 4)	4.09 (3.16 to 5.1)	20 (15 to 27)	14.53 (10.5 to 19.26)	4.577 (3.831 to 5.329)
Samoa	23 (18 to 30)	25.5 (19.77 to 32.55)	38 (28 to 52)	25.25 (19.13 to 34.29)	–0.041 (–0.169 to 0.088)
San Marino	17 (14 to 22)	52.65 (41.44 to 65.17)	30 (18 to 46)	50.75 (30.26 to 79.13)	–0.114 (–0.201 to –0.026)
Sao Tome and Principe	1 (1 to 2)	1.59 (1.02 to 2.24)	4 (3 to 7)	4.27 (2.59 to 6.69)	3.521 (3.232 to 3.811)
Saudi Arabia	293 (184 to 432)	4.73 (2.96 to 6.99)	3203 (2226 to 4410)	15.03 (10.97 to 19.74)	4.097 (3.877 to 4.317)
Senegal	198 (128 to 276)	5.88 (3.82 to 8.09)	578 (387 to 821)	7.45 (5.06 to 10.38)	0.773 (0.146 to 1.405)
Serbia	5646 (4706 to 6701)	46.14 (38.57 to 54.56)	11693 (8874 to 15199)	75.46 (56.66 to 99.15)	1.74 (1.472 to 2.009)
Seychelles	13 (10 to 17)	23.87 (17.74 to 29.94)	34 (26 to 43)	29.29 (22.91 to 36.75)	0.682 (0.262 to 1.103)
Sierra Leone	139 (88 to 197)	7.21 (4.58 to 10.14)	307 (189 to 447)	8.5 (5.27 to 12.3)	0.584 (0.395 to 0.773)
Singapore	560 (465 to 665)	24.48 (20.46 to 28.94)	1283 (1059 to 1537)	15.9 (13.17 to 19.03)	–1.515 (–1.789 to –1.239)

Slovakia	3346 (2877 to 3857)	55.83 (47.96 to 64.17)	4851 (3568 to 6416)	53.05 (39.11 to 70.11)	–0.235 (–0.545 to 0.077)
Slovenia	1298 (969 to 1743)	52.73 (39.28 to 70.82)	2199 (1634 to 3004)	54.9 (40.58 to 74.46)	0.018 (–0.206 to 0.242)
Solomon Islands	17 (11 to 26)	11.37 (7.47 to 16.53)	53 (32 to 78)	16.17 (10.51 to 23.38)	1.171 (1.018 to 1.324)
Somalia	116 (50 to 224)	4.44 (1.97 to 8.4)	239 (95 to 452)	3.45 (1.43 to 6.37)	–0.862 (–0.939 to –0.785)
South Africa	6059 (4636 to 7901)	28.38 (21.74 to 37.38)	9919 (7796 to 12301)	21.65 (17.23 to 26.76)	–0.759 (–0.989 to –0.529)
South Sudan	147 (83 to 255)	6.13 (3.54 to 10.49)	212 (114 to 360)	5.6 (3.11 to 9.15)	–0.321 (–0.413 to –0.229)
Spain	23449 (20380 to 26361)	44.42 (38.86 to 49.81)	42092 (36302 to 48450)	51.06 (43.96 to 58.51)	0.448 (0.313 to 0.584)
Sri Lanka	705 (477 to 939)	6.47 (4.51 to 8.54)	1656 (1035 to 2521)	6.26 (3.94 to 9.36)	–0.106 (–0.499 to 0.288)
Sudan	495 (278 to 908)	5.19 (2.92 to 9.46)	2583 (1516 to 4518)	13.7 (8.06 to 23.87)	3.386 (3.271 to 3.501)
Suriname	19 (15 to 24)	7.3 (5.81 to 8.9)	193 (145 to 254)	30.78 (23.19 to 40.32)	5.254 (4.579 to 5.932)
Sweden	8727 (7488 to 9941)	61.21 (52.83 to 69.17)	9225 (7742 to 10772)	45.64 (38.59 to 53.06)	–0.996 (–1.192 to –0.8)
Switzerland	3724 (3223 to 4285)	37.54 (32.59 to 43.08)	7605 (6508 to 8909)	46.32 (39.65 to 54.28)	0.687 (0.366 to 1.009)
Syrian Arab Republic	540 (380 to 737)	9.89 (6.94 to 13.37)	2163 (1525 to 3052)	16.36 (11.76 to 22.7)	1.72 (1.296 to 2.146)
Taiwan (Province of China)	4191 (3551 to 4786)	24.43 (20.74 to 27.86)	13581 (10191 to 18676)	33.89 (25.43 to 46.52)	1.2 (0.953 to 1.447)
Tajikistan	415 (282 to 603)	14.92 (9.96 to 21.82)	704 (531 to 922)	14.24 (10.66 to 18.97)	–0.184 (–0.774 to 0.409)
Thailand	6922 (5375 to 8628)	18.54 (14.67 to 22.94)	14481 (9827 to 20366)	13.95 (9.54 to 19.56)	–1.036 (–1.449 to –0.622)
Timor-Leste	21 (11 to 33)	6.99 (3.79 to 11)	88 (51 to 129)	10.57 (6.32 to 15.35)	1.456 (1.154 to 1.758)
Togo	88 (61 to 122)	7.09 (5 to 9.77)	372 (247 to 550)	10.12 (6.84 to 14.71)	1.233 (1.019 to 1.447)

Tokelau	0 (0 to 0)	12.09 (8.04 to 16.55)	0 (0 to 0)	18.1 (11.36 to 25.12)	1.399 (1.334 to 1.465)
Tonga	9 (6 to 12)	16.6 (11.31 to 22.06)	17 (11 to 24)	21.73 (14.62 to 30.73)	0.934 (0.455 to 1.414)
Trinidad and Tobago	51 (41 to 61)	6.05 (4.89 to 7.19)	356 (241 to 507)	18.6 (12.62 to 26.45)	4.096 (3.115 to 5.086)
Tunisia	546 (394 to 726)	10.64 (7.71 to 14.15)	2264 (1584 to 3196)	17.64 (12.43 to 24.84)	1.764 (1.584 to 1.943)
Turkey	13982 (10393 to 18734)	36.55 (27.47 to 48.35)	42317 (32637 to 54325)	46.46 (35.88 to 59.6)	0.774 (0.519 to 1.031)
Turkmenistan	126 (105 to 147)	6.24 (5.23 to 7.32)	580 (441 to 772)	13.49 (10.25 to 17.7)	2.884 (1.589 to 4.196)
Tuvalu	1 (1 to 1)	13.55 (9.67 to 17.91)	2 (1 to 3)	18.58 (12.55 to 25.69)	1.091 (1.036 to 1.146)
Uganda	288 (166 to 423)	4.42 (2.61 to 6.39)	1163 (730 to 1729)	8.36 (5.3 to 12.24)	2.233 (2.081 to 2.385)
Ukraine	21408 (16966 to 26810)	29.76 (23.38 to 37.46)	36282 (27785 to 45780)	51.12 (38.58 to 64.64)	2.229 (–0.052 to 4.561)
United Arab Emirates	155 (77 to 292)	29.33 (14.33 to 53.81)	3460 (1557 to 5901)	59.38 (29.23 to 92.42)	2.423 (1.999 to 2.848)
United Kingdom	60190 (53356 to 66618)	68.34 (60.7 to 75.4)	63946 (55624 to 73698)	53.56 (46.94 to 61.19)	–0.841 (–0.95 to –0.731)
United Republic of Tanzania	1068 (708 to 1473)	9.6 (6.44 to 13.02)	2958 (1848 to 4322)	12.17 (7.77 to 17.59)	0.856 (0.529 to 1.183)
United States of America	210069 (181665 to 237272)	67.91 (59.27 to 76.15)	316826 (271292 to 362961)	57.7 (49.79 to 65.73)	–0.547 (–0.782 to –0.312)
United States Virgin Islands	10 (7 to 12)	11.07 (8.28 to 14.07)	55 (40 to 74)	28.85 (21.15 to 38.59)	3.377 (3.156 to 3.597)
Uruguay	2347 (1989 to 2704)	60.63 (51.15 to 69.79)	3817 (3225 to 4444)	76.45 (64.43 to 89.08)	0.734 (0.424 to 1.045)
Uzbekistan	433 (314 to 595)	3.67 (2.68 to 5.05)	2667 (1977 to 3445)	11.44 (8.76 to 14.47)	4.117 (3.552 to 4.684)
Vanuatu	5 (3 to 7)	7.09 (4.65 to 10.23)	15 (10 to 22)	8.78 (5.66 to 12.54)	0.726 (0.538 to 0.914)
Venezuela (Bolivarian Republic of)	802 (643 to 957)	8.27 (6.69 to 9.8)	5388 (3685 to 7573)	18.22 (12.51 to 25.48)	2.569 (1.052 to 4.108)
Viet Nam	2717 (1947 to 3613)	6.65 (4.79 to 8.82)	14590 (10161 to 20420)	15.19 (10.73 to 20.92)	2.862 (2.721 to 3.003)
Yemen	354 (188 to 598)	6.76 (3.61 to 11.29)	1507 (1082 to 2171)	10.98 (7.98 to 15.63)	1.7 (1.551 to 1.849)

Zambia	227 (143 to 330)	8.35 (5.4 to 11.91)	725 (434 to 1152)	11.53 (7 to 17.76)	1.142 (1.068 to 1.215)
Zimbabwe	832 (651 to 1037)	20.59 (16.37 to 25.43)	1556 (1091 to 2102)	22.66 (16.23 to 30.07)	0.407 (0.148 to 0.666)

DALY: disability-adjusted life-year; AAPC: average annual percentage change.
